# Developmental exposure of California mice to endocrine disrupting chemicals and potential effects on the microbiome-gut-brain axis at adulthood

**DOI:** 10.1038/s41598-020-67709-9

**Published:** 2020-07-02

**Authors:** Sarabjit Kaur, Saurav J. Sarma, Brittney L. Marshall, Yang Liu, Jessica A. Kinkade, Madison M. Bellamy, Jiude Mao, William G. Helferich, A. Katrin Schenk, Nathan J. Bivens, Zhentian Lei, Lloyd W. Sumner, John A. Bowden, Jeremy P. Koelmel, Trupti Joshi, Cheryl S. Rosenfeld

**Affiliations:** 1 Christopher S Bond Life Sciences Center, University of Missouri, Columbia, MO 65211 USA; 2 Biomedical Sciences, University of Missouri, Columbia, MO 65211 USA; 3 MU Metabolomics Center, University of Missouri, Columbia, MO 65211 USA; 4 MU Institute of Data Science and Informatics, University of Missouri, Columbia, MO 65211 USA; 5 Food Science and Human Nutrition, University of Illinois, Urbana, IL 61801 USA; 6 Physics, Randolph College, Lynchburg, VA 24503 USA; 7 DNA Core Facility, University of Missouri, Columbia, MO 65211 USA; 8 Department of Biochemistry, University of Missouri, Columbia, MO 65211 USA; 9 Department of Physiological Sciences, College of Veterinary Medicine, University of Florida, Gainesville, FL 32610 USA; 10 Center for Environmental and Human Toxicology, College of Veterinary Medicine, University of Florida, Gainesville, FL 32610 USA; 11 Environmental Health Sciences, Yale University, New Haven, CT 06510 USA; 12 Department of Health Management and Informatics, School of Medicine, University of Missouri, Columbia, MO 65211 USA; 13 Thompson Center for Autism and Neurobehavioral Disorders, University of Missouri, Columbia, MO 65211 USA; 14 Genetics Area Program, University of Missouri, Columbia, MO 65211 USA

**Keywords:** Endocrinology, Data integration, Bioinformatics, Model vertebrates, Metabolomics, Neurology, Neurological disorders

## Abstract

Xenoestrogens are chemicals found in plant products, such as genistein (GEN), and in industrial chemicals, e.g., bisphenol A (BPA), present in plastics and other products that are prevalent in the environment. Early exposure to such endocrine disrupting chemicals (EDC) may affect brain development by directly disrupting neural programming and/or through the microbiome-gut-brain axis. To test this hypothesis, California mice (*Peromyscus californicus*) offspring were exposed through the maternal diet to GEN (250 mg/kg feed weight) or BPA (5 mg/kg feed weight, low dose- LD or 50 mg/kg, upper dose-UD), and dams were placed on these diets two weeks prior to breeding, throughout gestation, and lactation. Various behaviors, gut microbiota, and fecal metabolome were assessed at 90 days of age. The LD but not UD of BPA exposure resulted in individuals spending more time engaging in repetitive behaviors. GEN exposed individuals were more likely to exhibit such behaviors and showed socio-communicative disturbances. BPA and GEN exposed females had increased number of metabolites involved in carbohydrate metabolism and synthesis. Males exposed to BPA or GEN showed alterations in lysine degradation and phenylalanine and tyrosine metabolism. Current findings indicate cause for concern that developmental exposure to BPA or GEN might affect the microbiome-gut-brain axis.

## Introduction

Many fetuses and infants are exposed to xenoestrogens, such as bisphenol A (BPA) and genistein (GEN), through the placenta and milk^1–4^. Infants that are fed soy-based diets may also be directly exposed to phytoestrogens, in particular GEN. Developmental exposure to such endocrine disrupting chemicals (EDC) might additionally disrupt normal sex-dependent child behaviors and increase the risk for autism spectrum disorders (ASD) and other neurobehavioral disorders^5–13^.

We previously tested the effects of BPA in two *Peromyscus* species. This genus of rodents is distributed solely throughout North America. The first species of *Peromyscus* we tested was deer mice (*P. maniculatus baidii*), which are polygynous and exhibit only maternal care. Males of this species are highly dependent upon spatial navigation to locate widely dispersed prospective female breeding partners^14^. Developmental exposure to BPA or ethinyl estradiol (EE, estrogen of birth control pills) suppressed spatial navigational learning and memory in male deer mice, and females exposed to LD BPA or EE exhibited a masculinized pattern for this behavioral trait, as evidenced by enhanced spatial learning and memory^15,16^. Next, we examined effects of developmental exposure to BPA on California mice (*P. californicus*), who in contrast to deer mice evolved monogamous pair bonding behavior and biparental care^17^. Early BPA exposure resulted in decreased territorial marking behavior by male California mice, and exposed male and female California mice exhibited reduced parental care^18,19^. We have recently shown that California mice developmentally exposed to GEN show at weaning impairments in socio-communication behaviors^20^.

While developmental exposure to BPA and GEN may lead to later neurobehavioral disruptions by directly affecting neural programming, it is also clear that these chemicals can induce changes in the gut microbiota composition, which in turn can impact the microbiome-gut-brain axis^21^. Past rodent model studies testing the effects of perinatal BPA diet exposure have shown sex- and generational-dependent differences in gut microbiota compositions result when compared to those derived from dams maintained on a control, phytoestrogen-free diet^22^. Colic-liver inflammation has been shown to be induced by perinatal BPA diets in rabbits, leading to altered colon, cecum, and fecal microbiome profiles^23^. Perinatal BPA exposure of mice results in gut dysbiosis and immune system changes that precede the onset of obesity^24^. Other studies with dogs, rodent models, and zebrafish provide further evidence that BPA affects gut bacterial populations^25–29^.

Previous experiments have shown that varying connections of GEN-enriched or soy-based diets alter the gut microbiome in rodents and humans^30–37^. Soy formula diets in pigs are linked to changes in intestinal epithelial lining, resident microbes, and anti-inflammatory marker alterations^38^. Mouse model studies using direct dietary GEN exposure in the pre- or post-natal period are associated with gut microbiome changes, which were in turn correlated with changes in the metabolome and cognitive functioning^39–41^.

There is increasing evidence that the gut microbiome can exert neurobehavioral effects on the host via the microbiome-gut-brain axis with bacterial metabolites being potential mediators of such responses^21,42^. Past studies of urine metabolome analysis of children with autism spectrum disorders (ASD) displayed significantly altered metabolite profiles, including decreased levels of antioxidants, suggesting increased vulnerability to oxidative stress. In our recent study, we found that pre- and post-natal exposure of male and female California mice to GEN resulted at weaning in several gut microbiota and metabolome changes, and these changes were associated with disruptions in socio-communication behaviors^20^. However, it is not clear if developmental exposure to GEN and BPA can result in persistent gut microbiota and metabolite changes that are associated with potential socio-communication deficits and other behavioral traits at adulthood that replicate those observed in autistic children.

To address these critical knowledge gaps, we placed California mice dams for two weeks prior to breeding on diets containing a lower dose BPA (LD BPA), upper dose BPA (UD BPA), GEN, or a phytoestrogen-free control diet. Females were maintained on these diets throughout gestation and lactation. When male and female offspring reached adulthood (90 days of age), we tested them in a suite of behavioral tests designed to measure spatial learning and memory, anxiety-like and exploratory behaviors, and socio-communication. At this time, fecal samples were collected for gut microbiota and metabolome analyses, which were then correlated to the behavioral results. The underlying hypothesis tested in the current studies is that early exposure to BPA or GEN may lead to longstanding behavioral disturbances, some of which may be linked to gut microbiota/metabolome changes.

## Results

The general experimental design is depicted in Supplementary Fig. 1.

### Behaviors

#### Barnes maze

To determine whether perinatal exposure to BPA or GEN affects spatial learning and memory^15,16,18,43^, California mice offspring were tested beginning at 90 days of age in the Barnes maze. The primary behavioral category altered by perinatal treatment in this maze was latency to find the correct escape hole (p = 0.02). As no perinatal treatment × sex interactive effects were noted, results from both sexes within each group were combined and analyzed together. GEN exposed individuals showed reduced latency time to enter the correct hole compared to AIN (control) counterparts (Supplementary Fig. 2A). While no differences in latency time were observed in the LD BPA group, they spent increased time in the correct zone relative to AIN individuals (Supplementary Fig. 2B). GEN exposed individuals showed increased speed relative to AIN individuals (Supplementary Fig. 2C). While no perinatal treatment × sex interactive effects were noted for these behavioral categories, these results are included in Supplementary Table 1.

#### Social testing

**Figure 1:**
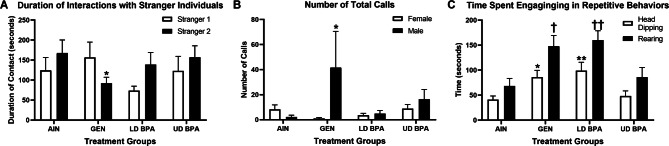
Socio-communication and EPM results. (**A**) Duration of interactions with Stranger mice. As shown, in trial 2, GEN exposed individuals spent less time interacting with a Stranger 2 mouse than AIN counterparts. *p = 0.01. (**B**) When tested in isolation, GEN exposed individuals called more in isolation. *p = 0.02. (**C**) When tested in the EPM, GEN and LD BPA individuals were more likely to engage in repetitive behaviors, including head-dipping and rearing. *p = 0.01, **p ≤ 0.0001, ^†^p = 0.0001, ^††^ p = 0.0007.

Crawley’s three-chambered social test was used to examine for potential EDC-induced social deficits^20,44^ Perinatal treatment affected the time the test individual spent with Stranger 1 vs. Stranger 2 in the third trial (p ≤ 0.05). GEN exposed individuals spent less time with Stranger 2 individuals compared to AIN individuals (Fig. 1A), suggestive of reduced social behaviors. Neither the LD BPA nor UD BPA altered this behavior. While perinatal treatment X sex interactions were not different (p > 0.05), these data are included in Supplementary Table 2).

#### Communication behaviors

We then tested ultrasonic and audible vocalizations produced in isolation. Perinatal treatment × sex interactive effects were observed for number of total calls (p = 0.003). GEN exposed males elicited greater number of calls than AIN males (Fig. 1B). Neither the LD BPA nor UD BPA affected the number of calls or other vocalization parameters.

#### Elevated plus maze (EPM)

Lastly, California mice offspring were tested in the elevated plus maze (EPM), which measures anxiogenic, exploratory, and repetitive behaviors. While no perinatal treatment differences were observed for time spent in open or closed arms, repetitive behaviors, including head-dipping and rearing, were affected by perinatal treatment (p ≤ 0.01). LD BPA and GEN exposed individuals spent more time engaging in both of these repetitive behaviors (p ≤ 0.05; Fig. 1C). While no significant differences were evident based on perinatal treatment and sex, these date are listed in Supplementary Table 2.

### Gut microbiota

All of the raw gut microbiota data are available on NCBI (BioProject number PRJNA577929, https://dataview.ncbi.nlm.nih.gov/object/PRJNA577929?reviewer=o9j3aooh017qu12rr7r0h2s9n8). None of the perinatal treatments affected overall α-diversity as determined by Chao1 and Shannon analyses (Supplementary Fig. 3A,B). Likewise, none of these treatments affected overall β-diversity as shown in the bar graphs illustrating the operational taxonomic units (OTU) or principal component analysis (PCA) diagram (Supplementary Fig. 3C,D). However, LEfSe analysis^45^ revealed that select gut bacteria were altered by the interaction of perinatal treatment and offspring sex.

**Figure 2:**
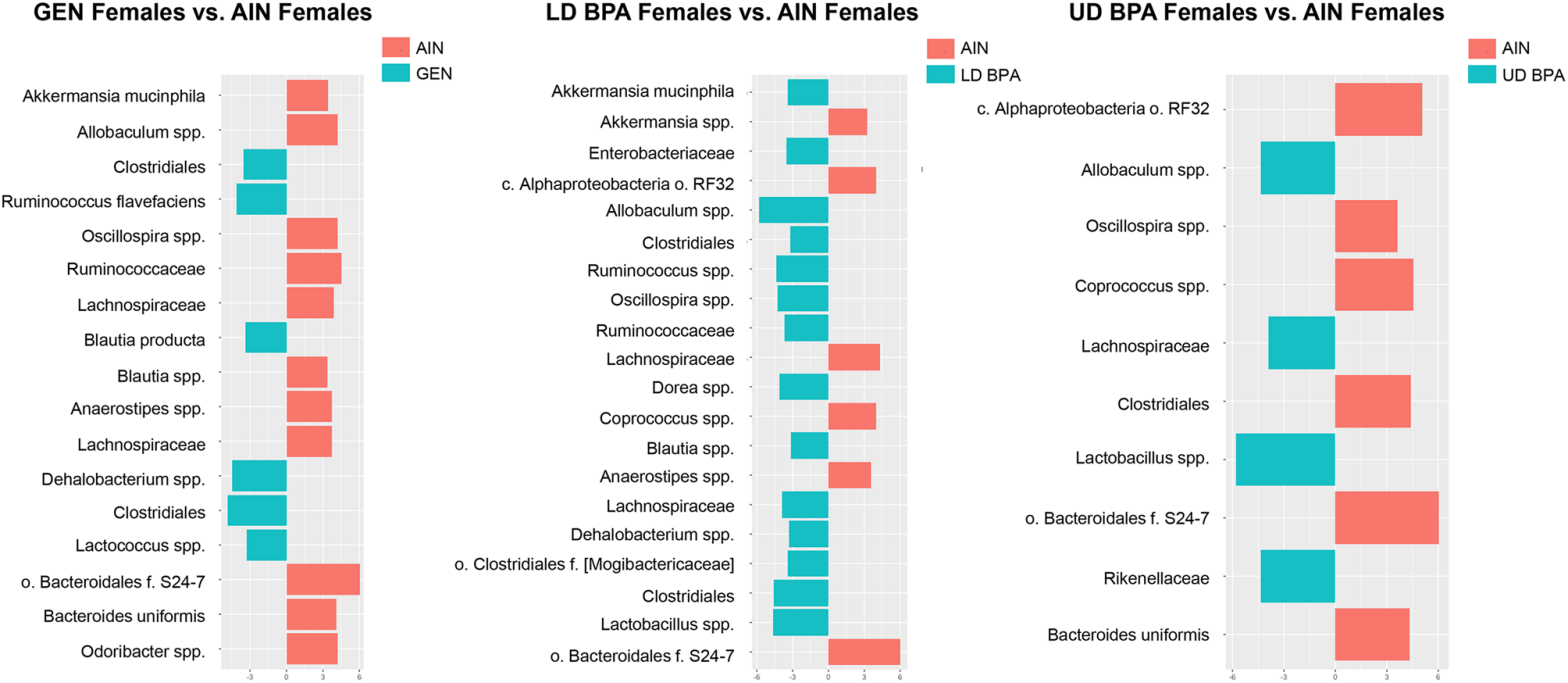
LEfSe gut bacterial results for female groups. (**A**) GEN females vs. AIN females. (**B**) LD BPA females vs. AIN females. (**C**) UD BPA females vs. AIN females. Those bacteria with an orange bar are greater in the treatment groups, whereas bacteria greater in AIN group are depicted with a blue bar.

In GEN females, *Ruminococcus flavefaciens*, *Blautia producta*, *Dehalobacterium *spp., *Clostridiales*, and *Lactococcus *spp. were increased relative to AIN control females (Fig. 2A). In contrast, *Akkermansia muciniphila*, *Allobaculum *spp., *Oscillospira *spp., Ruminococcaceae, *Blautia *spp., *Anaerostipes *spp., *Lachnospiraceae*, *Odoribacter *spp., *Bacteroidales* f.S24-7, and *Bacteroides uniformis* were decreased in GEN females relative to AIN females (Fig. 2A). In LD BPA females, *Blautia *spp., *Clostridiales*, *Dehalobacterium *spp., [*Mogibacteriaceae*], *Akkermansia muciniphila*, *Enterobacteriaceae*, *Ruminococcaceae*, *Lachnospiraceae*, *Dorea *spp., *Oscillospira *spp., *Ruminococcus *spp., *Clostridiales*, *Lactobacillus *spp., and *Allobaculum *spp. were increased relative to AIN females (Fig. 2B). *Akkermansia *spp., *Anaerostipes *spp., *Alphaproteobacteria* RF32, *Coprococcus *spp., *Lachnospiraceae*, and *Bacteroidales* f.S24-7 were reduced in LD BPA vs. AIN females (Fig. 2B). In UD BPA females, *Lachnospiraceae*, *Allobaculum *spp., *Rikenellaceae*, and *Lactobacillus *spp. were increased relative to AIN females (Fig. 2C). *Oscillospira *spp., *Bacteroides uniformis*, Clostridiales, *Coprococcus *spp., *Alphaproteobacteria* RF32, and *Bacteroidales* f.S24-7 were decreased in UD BPA vs. AIN females (Fig. 2C).

**Figure 3:**
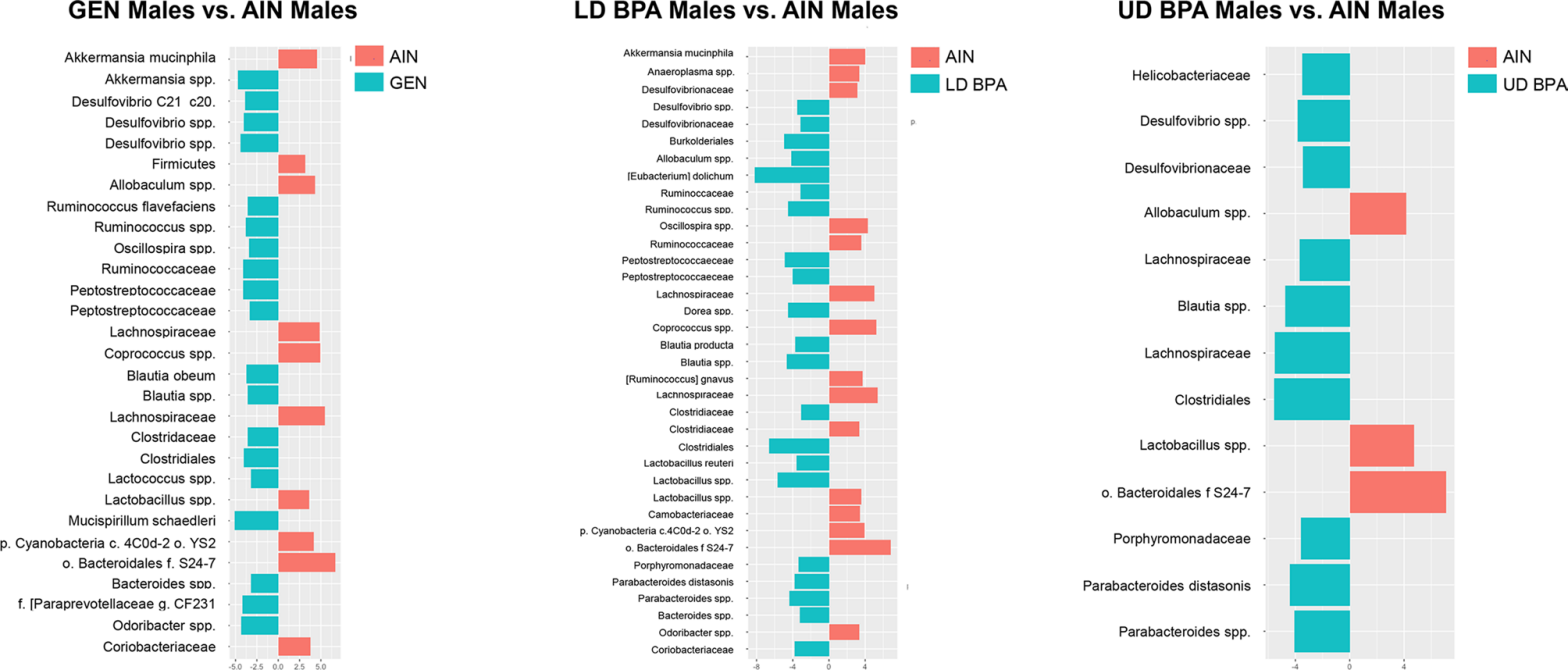
LEfSe gut bacterial results for male groups. (**A**) GEN males vs. AIN males. (**B**) LD BPA males vs. AIN males. (**C**) UD BPA males vs. AIN males. Those bacteria with an orange bar are greater in the treatment groups, whereas bacteria greater in AIN group are depicted with a blue bar.

In GEN males, *Lactococcus *spp., *Bacteroides *spp., *Peptostreptococcaceae*, *Oscillospira *spp., *Blautia *spp., *Ruminococcus flavefaciens*, Clostridiaceae, *Ruminococcus *spp., *Desulfovibrio; C21_c20*, *Clostridiales*, *Desulfovibrio *spp., Peptostreptococcaceae, *Ruminococcaceae*, [*Paraprevotellaceae*] CF231, *Odoribacter *spp., *Desulfovibrio *spp., *Akkermansia *spp., and *Mucispirillum schaedleri* were increased relative to AIN males (Fig. 3A). In contrast, *Firmicutes*, *Lactobacillus *spp., Coriobacteriaceae, *Cyanobacteria* c.4C0d-2 o.YS2, *Allobaculum *spp., *Akkermansia muciniphila*, *Lachnospiraceae*, *Coprococcus *spp., and *Bacteroidales* f.S24-7 were reduced in GEN vs. AIN males (Fig. 3A).

LD BPA males had relative increased amounts of *Clostridiaceae*, *Desulfovibrionaceae*, *Ruminococcaceae*, *Bacteroides *spp., *Porphyromonadaceae*, *Desulfovibrio *spp., *Lactobacillus; reuteri*, *Blautia producta*, *Coriobacteriaceae*, *Parabacteroides distasonis*, *Peptostreptococcaceae*, *Allobaculum *spp., *Parabacteroides *spp., *Ruminococcus *spp., *Dorea *spp., *Blautia *spp., *Peptostreptococcaceae*, *Burkholderiales*, *Lactobacillus *spp., *Clostridiales*, and [*Eubacterium*] *dolichum* relative amounts compared to AIN males (Fig. 3B). *Desulfovibrionaceae*, *Odoribacter *spp., *Clostridiaceae*, *Anaeroplasma *spp., *Carnobacteriaceae*, *Lactobacillus *spp., *Ruminococcaceae*, *Cyanobacteria* c.4C0d-2 o.YS2, *Akkermansia muciniphila*, *Oscillospira *spp., *Lachnospiraceae*, *Coprococcus *spp., *Lachnospiraceae*, and *Bacteroidales* S24-7 were reduced in LD BPA vs. AIN males (Fig. 3B). In UD BPA males, *Desulfovibrionaceae*, *Helicobacteraceae*, *Porphyromonadaceae, Lachnospiraceae*, *Desulfovibrio *spp., *Parabacteroides *spp., *Parabacteroides distasonis*, *Blautia *spp., *Lachnospiraceae*, and *Clostridiales* were increased compared to AIN males (Fig. 3C). *Allobaculum *spp., *Lactobacillus *spp., and *Bacteroidales* f.S24-7 were reduced in UD BPA vs. AIN males (Fig. 3C).

### Venn diagram comparison of bacterial differences

To examine for potential overlap amongst the groups in terms of bacterial changes, Venn diagram comparisons were done. Comparison of bacteria that were increased in GEN, LD BPA, and UD BPA females relative to AIN females, however, revealed no bacteria overlapped in all three groups (Supplementary Fig. 4A). However, two overlapped in GEN and LD BPA (*Dehalobacterium *spp., *Clostridiales*) and three overlapped in LD BPA and UD BPA (*Lachnospiraceae*, *Allobaculum *spp., and *Lactobacillus *spp.). In all three female groups, a Bacteroidales was reduced (Supplementary Fig. 4B). GEN and LD BPA females had two bacteria in common that were reduced (*Anaerostipes *spp. and *Lachnospiraceae*). Comparison of GEN and UD BPA females revealed two bacteria (*Oscillospira *spp. and *Bacteroides uniformis*) that showed similar changes. LD BPA and UD BPA likewise had two bacteria reduced in both groups relative to AIN (*Coprococcus *spp. and *Alphaproteobacteria* RF32).

Venn diagram comparison of bacteria increased in all three treatment male groups revealed that *Clostridiales*, *Blautia *spp., and *Desulfovibrio *spp. were elevated in all three relative to AIN males (Supplementary Fig. 4C). These five bacteria were increased in GEN and LD BPA males: *Bacteroides *spp., *Peptostreptococcaceae*, *Clostridiaceae*, *Peptostreptococcaceae*, and *Ruminococcaceae*, and the four bacteria increased in LD BPA and UD BPA include: *Desulfovibrionaceae*, *Porphyromonadaceae*, *Parabacteroides distasonis,* and *Parabacteroides *spp. Similar to the female treatment groups, a *Bacteroidales* was reduced in all treated male groups (Supplementary Fig. 4D). The four bacteria reduced in both GEN and LD BPAwere Cyanobacteria c.4C0d-2 o.YS2, *Akkermansia muciniphila*, *Lachnospiraceae*, and *Coprococcus *spp. The two reduced in GEN and UD BPA include: *Allobaculum *spp. and *Lactobacillus *spp.

### Metabolome

All of the raw metabolome data are available at the NIH Common Fund's National Metabolomics Data Repository (NMDR) website, the Metabolomics Workbench: current project ID is PR000932, and DOI number is 10.21228/M8710D. The weblink for the data is https://dev.metabolomicsworkbench.org:22222/data/DRCCMetadata.php?Mode=Project&ProjectID=PR000932&access=LduV2970.

GC–MS polar, GC–MS non-polar, and LC–MS/MS analyses were performed to obtain as much coverage in terms of profiling and identification of primary and specialized metabolites that differed between the various groups. Each of these approaches has their advantages and disadvantages. While LC–MS/MS has the ability to detect more molecular and underivatized features, many of these are currently not structurally identified. GC–MS polar is useful in profiling and identifying carbohydrates, amino acids, carboxy acids, alcohols, amines, and nucleotides. GC–MS non-polar is used to identify lipophilic metabolites such as fatty acids and sterols.

### GC–MS of polar fraction

**Figure 4:**
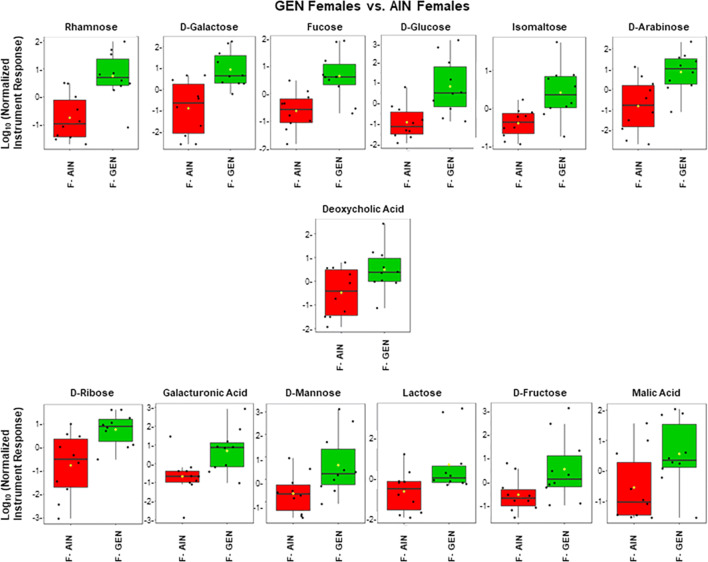
GC–MS polar fraction results for GEN females vs. AIN females. Results for known metabolites that differ in this comparison are shown with box and whisker plots, which show the upper and lower quartiles. The median is designated with the vertical line inside the box. The yellow dot indicates the mean value. Lines outside indicate the lowest and highest observations. Each replicate is shown as a single black dot. Graphs were generated with the MetaboAnalyst Program v. 4.0 (https://www.metaboanalyst.ca/).

**Figure 5:**
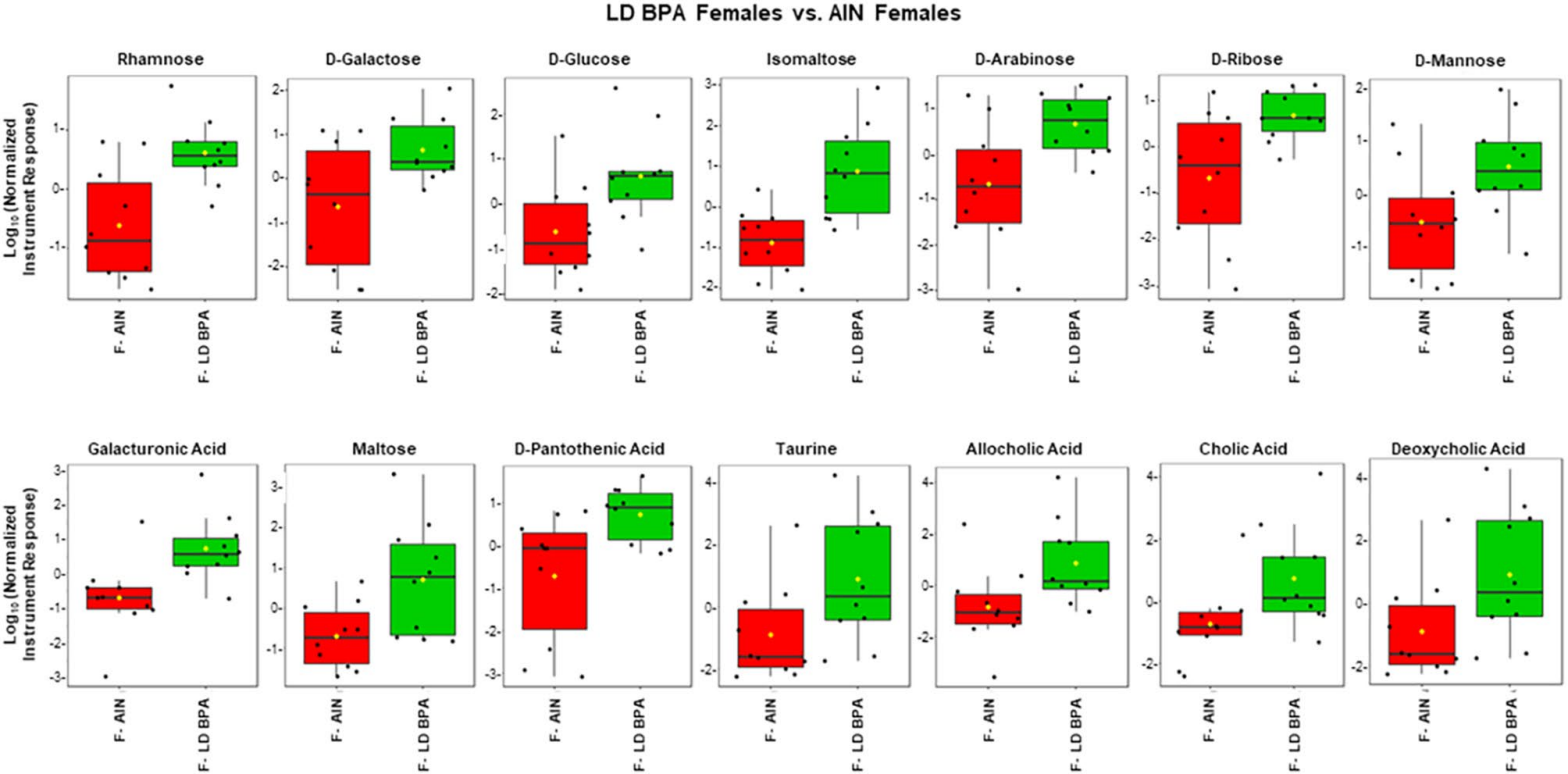
GC–MS polar fraction results for LD BPA females vs. AIN females. Results for known metabolites that differ in this comparison are shown with box and whisker plots, which show the upper and lower quartiles. The median is designated with the vertical line inside the box. The yellow dot indicates the mean value. Lines outside indicate the lowest and highest observations. Each replicate is shown as a single black dot. Graphs were generated with the MetaboAnalyst Program v. 4.0 (https://www.metaboanalyst.ca/).

**Figure 6:**
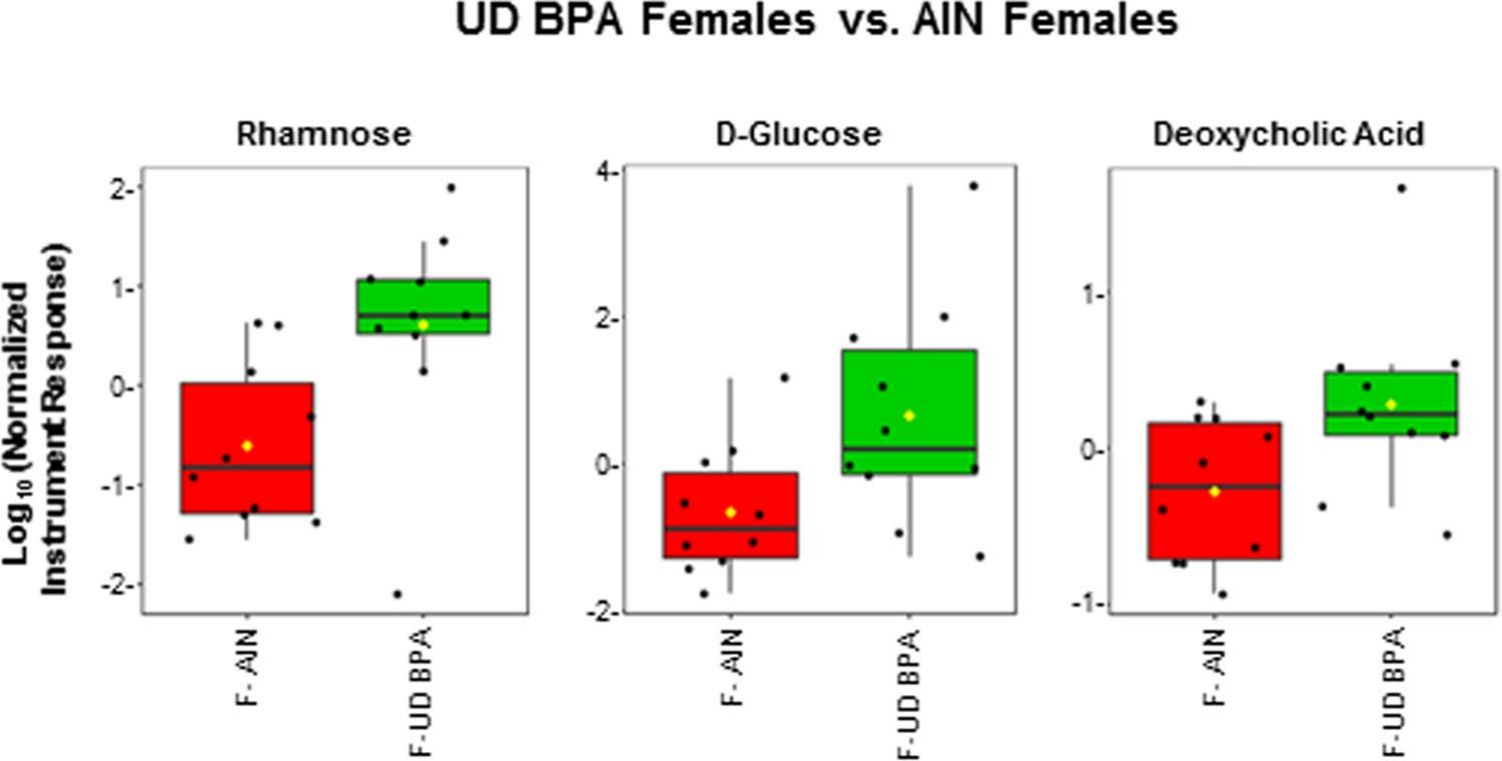
GC–MS polar fraction results for UD BPA females vs. AIN females. Results for known metabolites that differ in this comparison are shown with box and whisker plots, which show the upper and lower quartiles. The median is designated with the vertical line inside the box. The yellow dot indicates the mean value. Lines outside indicate the lowest and highest observations. Each replicate is shown as a single black dot. Graphs were generated with the MetaboAnalyst Program v. 4.0 (https://www.metaboanalyst.ca/).

**Figure 7:**
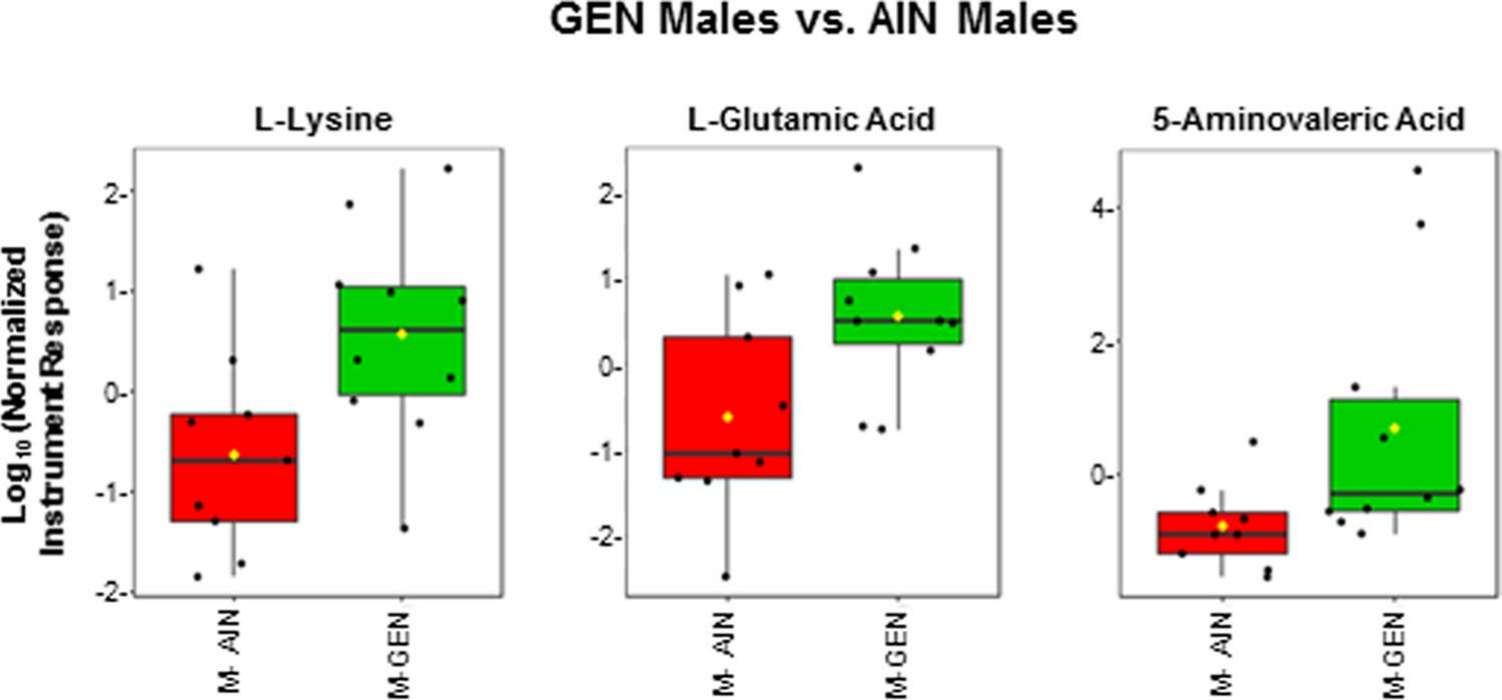
GC–MS polar fraction results for GEN males vs. AIN males. Results for known metabolites that differ in this comparison are shown with box and whisker plots, which show the upper and lower quartiles. The median is designated with the vertical line inside the box. The yellow dot indicates the mean value. Lines outside indicate the lowest and highest observations. Each replicate is shown as a single black dot. Graphs were generated with the MetaboAnalyst Program v. 4.0 (https://www.metaboanalyst.ca/).

**Figure 8:**
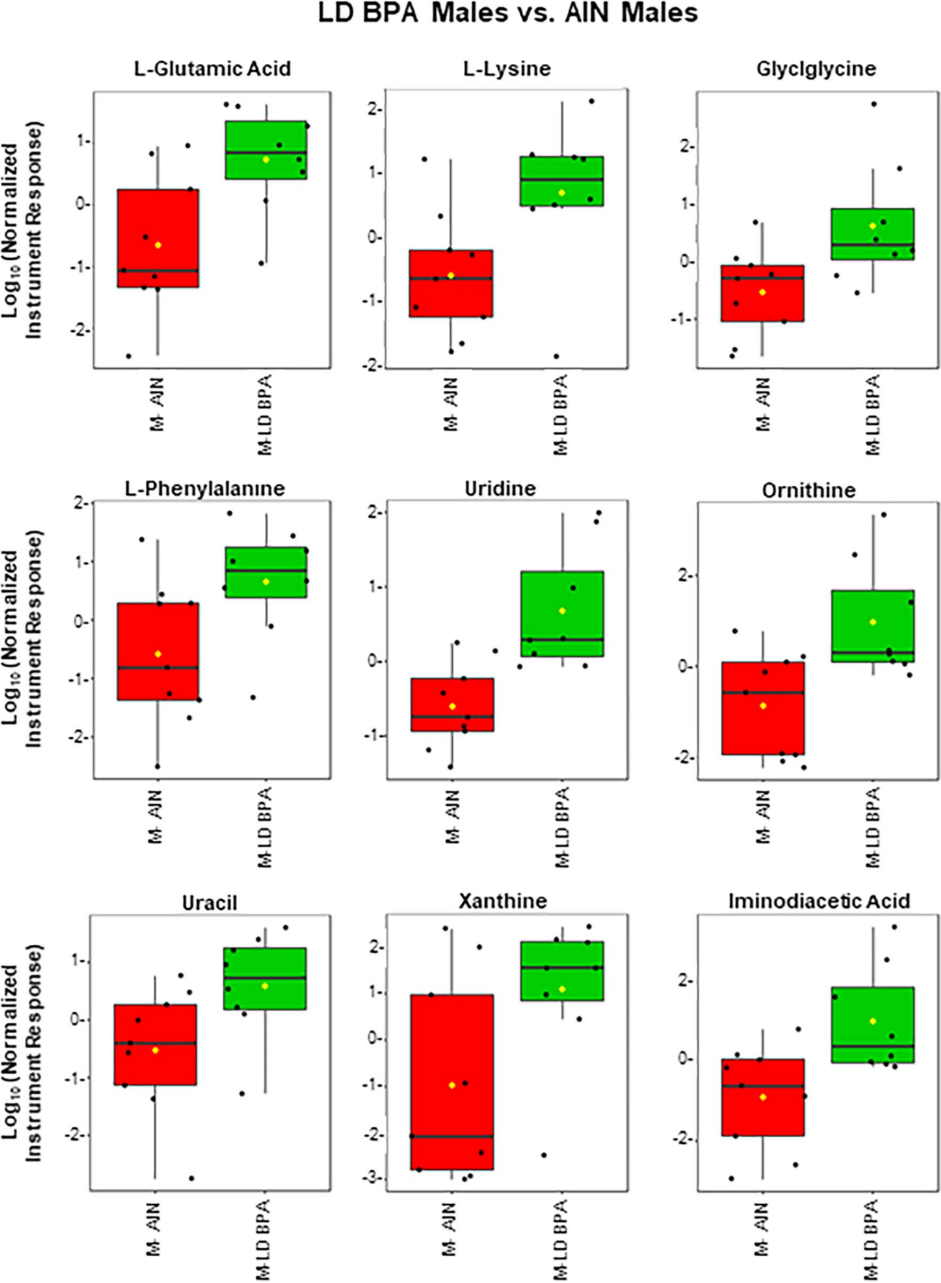
GC–MS polar fraction results for LD BPA males vs. AIN males. Results for known metabolites that differ in this comparison are shown with box and whisker plots, which show the upper and lower quartiles. The median is designated with the vertical line inside the box. The yellow dot indicates the mean value. Lines outside indicate the lowest and highest observations. Each replicate is shown as a single black dot. Graphs were generated with the MetaboAnalyst Program v. 4.0 (https://www.metaboanalyst.ca/).

The full list of metabolites identified with GC–MS polar fraction is included in Supplementary File 1. Volcano plots showing the number of significant metabolites relative to AIN counterparts is shown in Supplementary Fig. 5. Comparison of GEN vs. AIN females with this method revealed that several carbohydrates were increased in GEN females, including rhamnose, d-galactose, fucose, d-glucose, isomaltose, d-arabinose, d-ribose, galacturonic acid, d-mannose, lactose, d-fructose, and malic acid (Fig. 4). Additionally, the bile acid, deoxycholic acid was elevated in this group. Similarly, LD BPA females showed increased amounts of several sugars, including rhamnose, d-galactose, d-glucose, isomaltose, d-arabinose, d-ribose, galacturonic acid, d-mannose, and malose monohydrate (Fig. 5). Besides deoxycholic acid, allocholic acid and cholic acid were elevated for bile acids in LD BPA. Pantothenic acid was also elevated in this group. Rhamose, d-glucose and deoxycholic acid were similarly elevated in UD BPA females vs. AIN females (Fig. 6).

l-lysine, l-glutamic acid, and valeric acid were elevated in GEN males vs. AIN males (Fig. 7). Nine known metabolites, l-glutamic acid, l-lysine, glycglycine, l-phenylalanine, uridine, ornithine, uracil, xanthine, and iminodiacetic acid, were greater in LD BPA males relative to AIN males (Fig. 8). No known metabolites, however, differed between UD BPA and AIN males.

### GC–MS of non-polar fraction

The full list of metabolites identified with GC–MS non-polar fraction is provided in Supplementary File 2. Volcano plots showing the number of significant metabolites relative to AIN counterparts is detailed in Supplementary Fig. 6. Based on GC–MS non-polar analysis, six metabolites differed significantly between GEN females vs. AIN females (Supplementary Fig. 7). Known metabolites in this comparison include 3β, 5β-cholestan-3-ol and deoxycholic acid, which were increased in GEN females, whereas, 1-hexadecanol and n-heptadecanol were reduced in this group relative to AIN females. Four metabolites differed between LD BPA vs. AIN females (Supplementary Fig. 10). Of these, the only known metabolite was eicosatetraenoic acid, which was increased in LD BPA females. No known metabolites differed in UD BPA vs. AIN females (Supplementary Fig. 7). Six metabolites were different in GEN males vs. AIN males (Supplementary Fig. 7). For the metabolites currently known, eicosatetraenoic acid was increased in GEN males, but hexadecane-1,2-diol, 3β, 5β-cholestan-3-ol, and chenodeoxycholic acid were reduced in this group. Ten metabolites differed between LD BPA vs. AIN males (Supplementary Fig. 7). For the known metabolites, 2-monomyristin was increased but n-heptadecanol was reduced in LD BPA males relative to AIN males. Only one unknown metabolite was increased in UD BPA males relative to AIN males (Supplementary Fig. 7**)**.

### LC–MS/MS

The full list of metabolites identified with LC–MS/MS is provided in Supplementary File 3. Volcano plots revealing the number of significant metabolites relative to AIN counterparts is shown in Supplementary Fig. 8. While several metabolites differed between GEN vs. AIN females, the only one that could be identified was guanine, which was upregulated in the GEN group (Supplementary Fig. 9). In LD BPA females vs. AIN females, several metabolites differed, but the only known metabolite was lysophosphatidylethanolamine (LysoPE-15:0), which was decreased in the former group (Supplementary Fig. 9). Several metabolites differed between UD BPA vs. AIN females. Of the known metabolites, 3-hydroxy-7-ketolithocholic acid was increased in the UD BPA group; whereas, naringenin and eriodictyol were decreased in this group (Supplementary Fig. 9). In GEN and LD BPA males, 3,12-diol-7-one-5-cholanic acid was increased relative to AIN males (Supplementary Fig. 9). Additionally, 5-methyl-5-thioadenosine and citrulline were increased in LD BPA males compared to AIN males (Supplementary Fig. 9). Other metabolites differed between treated male groups and AIN controls, but we were not able to identify them using a variety of approaches, as detailed in the Supplementary Methods section.

### Metabolomics enrichment analyses

Based on all of the metabolites that differed with the three different analytical methods, enrichment analyst was done with the MetaboAnalyst program v. 4.0. As shown in Supplementary Fig. 10, the top five pathways enriched in GEN F vs. AIN F were galactose metabolism, lactose degradation, fructose and mannose degradation, lactose synthesis, and gluconeogenesis. In LD BPA vs. AIN females, the top five enriched pathways include lactose degradation, galactose metabolism, bile acid biosynthesis, sphingolipid metabolism, and taurine and hypotaurine metabolism (Supplementary Fig. 10). For UD BPA vs. AIN females, lactose degradation, glucose-alanine cycle, lactose synthesis, transfer of acetyl groups into mitochondria, and glycolysis were the primary pathways presumed to be affected in the former group (Supplementary Fig. 10). For male groups, there were insufficient number of known metabolites in UD BPA males vs. AIN males, to perform this analysis.

For GEN males vs. AIN males, the top pathways implicated were lysine degradation, biotin metabolism, arachidonic acid metabolism, malate-aspartate shuttle, and alanine metabolism (Supplementary Fig. 11). For the LD BPA males vs. AIN males, the main pathways that were likely to be affected include phenylalanine and tyrosine metabolism, urea cycle, lysine degradation, beta-alanine metabolism, and biotin metabolism (Supplementary Fig. 11).

### Multiomics integrative correlation analyses

Potential bacterial genera changes, fecal metabolome, and behavioral results were correlated by using mixOmics R package^46^ with an r value set to 0.70. As shown, several correlations existed for the various treatment groups and comparisons, but for GEN females vs. AIN females the circos plot analyses revealed no individual bacteria correlated with the other results (Supplementary Fig. 12). However, d-glucose and d-galactose inversely correlated with several vocalization behaviors, including average power above 20 kHz, i.e., in the ultrasonic range. N-heptadecanol and 1-hexadecanol positively correlated with time spent in the closed arms for the EPM.

For LD BPA females vs. AIN females, *Lachnospiraceae* and *Enterobacteriaceae* positively correlated with LysoPE (Supplementary Fig. 13). These two bacteria and *Akkermansia muciniphila* positively correlated with performance in the Barnes maze, such as latency, distance traveled, and speed. Lachnospiraceae positively correlated with the metabolite, erythritol. While the metabolites identified with LC–MS were correlated with vocalization behavior parameters, many of these significant metabolites are currently unidentified.

For UD BPA females vs. AIN females, *Bacteroidales* f.S24-7 positively correlated with several unknown metabolites identified with LC–MS (Supplementary Fig. 14). This bacterium also positively correlated with several parameters measured in the Barnes maze, including distance traveled, speed, and entries into correct hole.

For GEN males vs. AIN males comparison, many inter-relationships are evident in the circos plot analysis (Supplementary Fig. 15). Lysine, glutamic acid, and 5-aminovaleric acid positively correlated with the various social behaviors. Several unknown metabolites identified with LC–MS correlated with the vocalization behaviors. Chenodeoxycholic acid and hexadecane-1,2-diol inversely correlated with parameters measured in the Barnes maze, whereas 3β, 5β-cholestan-3-ol positively correlated with these behaviors.

For LD BPA males vs. AIN males, several bacteria, including *Ruminococcus *spp., *Burkholderiales*, *Blautia *spp., *Parabacteroides *spp., Peptostreptococcaceae, *Blautia producta*, *Clostridiaceae*, and *Dorea *spp. positively correlated with various metabolites identified with LC–MS, GC–MS polar, and GC–MS non-polar fractions, including 2-monomyristin, l-lysine, 2′deoxyinosine, glyclglycine, uridine, iminodiacetic acid, and 5-cholanic acid 3,12,diol (Supplementary Fig. 16).

For UD BPA males vs. AIN males, *Blautia *spp., *Parabacteroides *spp., *Helicobacteraceae*, *Parabacteroides distasonis*, *Desulfovibrio *spp., and Porphyromonadaceae positively correlated with various vocalization behaviors (Supplementary Fig. 17). In contrast, many of the unknown metabolites negatively correlated with these behavioral traits.

## Discussion

In this study, we sought to determine how developmental exposure to environmentally relevant doses of BPA and GEN affected potential expression of  autistic-like behaviors at adulthood. Additionally, effects of these chemicals on gut microbiota and gut metabolome were examined as such changes might impact the microbiome-gut-brain axis.

Conflicting results were obtained for the behavioral studies. California mice developmentally exposed to GEN showed some improvements for spatial learning and memory, as evidenced by their decreased latency over the five-day trial to enter the correct hole. It is not clear though whether such improvements were due to them being faster than their AIN control counterparts or actual cognitive improvements. Perinatal exposure of rats to GEN (1 or 10 mg/kg/day administered to the dam from gestational day 10 to postnatal day 14) improved spatial learning and memory but impaired passive avoidance learning and memory^47^. Other studies with adult rodent models further suggests that GEN improves spatial and placement learning and memory^48–51^. Conversely, another study showed that male rats exposed to GEN (5 mg/kg feed weight) through the maternal diet during both gestation and lactation exhibited spatial learning and memory deficits when tested in the Morris water maze, but those males exposed to GEN only during gestation or lactation did not show such changes^52^. The collective results suggest that the dose and timing of GEN exposure may lead to disparate effects on this behavioral domain.

For socio-communicative behaviors, GEN exposed individuals were less social, as evidenced by reduced time spent investigating the Stranger 2 individual when tested in a three-chamber social test. This group went on to exhibit greater number of vocalizations when placed in isolation, suggestive of potential increased anxiety. When we tested comparable California mice at weaning, GEN exposed females already started to show social deficits at 30 days of age^20^. Rats developmentally exposed to GEN, as administered to the dams as aglycone genistein, suppressed novel preference and open field exploration in females tested at PND 24–28^53^. In humans, soy infant formula consumption correlates with reduced female-typical play behavior in girls and increase risk of autistic behaviors^5,6^. Thus, several studies sugggest early exposure to GEN results in socio-communication deficits, especially in female offspring.

When tested in the EPM, it was predicted that EDC-treated groups would be more anxious as past studies indicate BPA and GEN increase anxiogenic behaviors^15,16,54^. Neither of these compounds resulted in increased anxiety-like behaviors in the current studies. Instead, California mice developmentally exposed to LD BPA and GEN showed increased stereotypical/repetitive behaviors, namely head-dipping and rearing. However, the UD BPA exposure did not induce such deficits. Other studies have noted only low dose and non-monotonic effects with this chemical^55,56^.

It was initially surprising to us that we did not see either the LD BPA or UD BPA affect other behaviors that we have previously shown to be vulnerable to these EDC. The most likely explanation is that developmental exposure to BPA and GEN may impact different behaviors in various species, especially those that are considered sexually selected traits^15,16,57^. In our previous studies, we found that BPA exposure suppressed territorial marking, which is a sexually selected trait in this species, but not spatial learning and memory in male California mice^18^. In both male and female California mice, we have previously demonstrated that developmental exposure to BPA reduced biparental care provided to their offspring^19^. These behaviors were not measured in the current studies. Our previous studies with related polygynous deer mice cousins demonstrated that early exposure to BPA suppressed spatial learning and memory in males^15,16^, who depend upon this behavior to locate potential mates who are randomly scattered throughout the environment. The LD BPA group of females had masculinization of this trait in that they demonstrated improved spatial learning and memory relative to control females^15,16^.

Besides inducing direct effects on the brain, developmental exposure to one or both doses of BPA or GEN may result in gut dysbiosis and thereby potentially inducing affects on the microbiome-gut-brain axis. In our previous studies, we showed that California mice developmentally exposed to the UD BPA or GEN show distinct signature patterns of gut microbiota changes at weaning^20,22^. The current studies sought to determine whether such EDC-induced gut bacterial changes identified at weaning persist to adulthood and whether additional bacteria changes might be detected.

We will consider select bacteria whose relative proportions were altered in one or more of the groups, as demonstrated previously. At weaning, the relative proportions of *Clostridiales* were increased in UD BPA females^22^, but in adults of this same treatment group, a bacterium within *Clostridiales* was decreased compared to AIN controls in current study. In LD BPA and GEN females, the relative proportion of this bacterium was, however, increased at adulthood.

*Bacteroidales* was the only bacteria whose relative proportions were decreased at weaning and adulthood in GEN males and females. In contrast, *Lactococcus *spp. was the single bacterium whose relative amounts were elevated in GEN females both at weaning and adulthood. In GEN males, both Lachnospiraceae and *Allobaculum *spp. were increased at weaning but were reduced in both groups by adulthood.

Similar to GEN males and females at both weaning and adulthood, *Bacteroidales* was decreased in males and females of UD BPA and LD BPA groups. Two-month-old male mice provisioned with a high sucrose diet also show reductions in *Bacteroidales* but increased amounts of *Clostridiales*, and both bacterial changes are related to compromised cognitive plasticity^58^. Relative amounts of *Clostridiales* are increased in all three EDC exposed male groups. *Blautia *spp., and *Desulfovibrio *spp. were also elevated in all three treated male groups. Patients with major depressive disorder demonstrate increased amounts of *Blautia *spp. amongst other bacterial changes^59,60^. Taken together, developmental exposure of California mice to BPA or GEN may result in what might be considered a signature stress “microbiome” profile.

Rabbits perinatally exposed to BPA (200 μg BPA/kg body weight orally administered to pregnant does from GD 15 through PND 15) had reductions in *Ruminococcaceae* and *Oscillospira *spp. in the case of those directly exposed to BPA or *Odoribacter *spp. in the case of BPA-exposed offspring^23^. *Oscillospira *spp. was decreased in California mice females developmentally exposed to the UD BPA. In contrast, relative amounts of this bacteria and *Ruminococcaceae* were elevated in LD BPA females. Males exposed to LD BPA had reductions in relative amounts of *Ruminococcaceae*, *Oscillospira *spp*.* and *Odoribacter *spp*.* Other studies show that exposure to phytoestrogens, including GEN, can alter other gut microbes^30–38,61,62^. The collective findings suggest that while some bacterial changes are identical across studies examining effects of GEN or BPA on gut microbiota, divergent results across studies may result from different EDC tested, dose and duration of the chemical exposure, window of exposure, host species examined, and sex of the individual.

As one of the ways in which gut bacterial changes may impact the microbiome-gut-brain axis is the through alteration of bacterial metabolites^21^, we examined the fecal metabolome profile in the different groups with the caveat that this high throughput cannot generally distinguish metabolites produced by the host from bacterial derived ones. We will consider a few of the example metabolites that were altered in one or more of the groups and potential metabolic pathways that might be affected based on the pattern of metabolic changes. Rhamnose was increased in GEN, LD BPA, and UD BPA females. *Blautia *spp*.* tends to ferment rhamnose to 1,2-propanediol^63^. Different *Blautia *spp*.* were increased in all three female treatment groups, suggesting increase rhamnose precursor stimulates growth of this bacteria that acts then upon this carbohydrate. GEN and LD BPA groups were the two groups where behavioral differences were observed relative to AIN controls. Interestingly, the greatest number of metabolite changes were found in these same groups, especially females exposed to GEN or LD BPA. In all three female groups, various sugars were increased with GEN and LD BPA having the greatest number of such metabolite changes that also closely mirrored each other. Enrichment analyses also revealed that synthesis and metabolism of various carbohydrates, such as galactose metabolism, lactose degradation, and lactose synthesis, were among the top pathways in all three treatment groups. Capillary electrophoresis time-of-flight mass spectrometry (CE–TOF/MS) metabolomics analysis of urine from rats treated with BPA revealed amino acids, polyamines, nucleosides, organic acids, carbohydrates, pterins, polyphenols, and sugar phosphates were found as the most significantly differential metabolites^64^. This study also found BPA-induced changes in several neurotransmitters (glutamate, gamma-aminobutyric acid, and noradrenaline) and neurotransmitter related metabolites (tyrosine, histamine, valine, and taurine).

Deoxycholic acid was upregulated in all three female treatment groups. Cholic acid, which was also increased in LD BPA females vs. AIN females, is converted by intestinal bacteria to deoxycholic acid^65,66^. One report with Alzheimer’s disease (AD) patients suggest that the primary bile acid is reduced but the secondary bile acid, deoxycholic acid, is elevated in such individuals and is strongly associated with cognitive decline, a result similar to serum and brain samples from another cohort group, Rush Religious Orders and Memory and Aging Project^67^.

In GEN males, l-lysine and its bacterial degradation product, 5-aminovaleric acid, were elevated. The latter metabolite may exhibit weak inhibitory activity on gamma aminobutyric acid B (GABAB) receptors^68^. Lysine degradation was one of the top pathways altered in GEN males vs. AIN males and LD BPA males vs. AIN males, suggestive that developmental exposure to certain doses of BPA may mirror the effects of GEN exposure. The urea cycle was another enriched pathway in both of these male groups. This cycle is essential for disposal of excess nitrogen through urea biosynthesis. High concentrations of ammonia or its metabolites can result in central nervous system disorders by altering glutamate transport by its transporters^69^. Glutamic acid (glutamate) was increased in both of these groups. This amino acid is essential in detoxifying ammonia but also acts as a primary excitatory neurotransmitter, which can be converted to glutamine or GABA. Glutamate and GABA neurotransmission have been implicated in mood disorders^70,71^.

MixOmics analysis revealed that in GEN females vs. AIN females, d-glucose and d-galactose were negatively correlated with ultrasonic vocalizations (USV). However, *Glut3* ± mice, who cannot transport glucose across neuronal cell membranes, show reduced glucose concentrations in the brain, have reduced vocalizations^72^. The total number of calls was increased in GEN vs. AIN individuals, but this includes those in the non-ultrasonic and ultrasonic range. Collectively, the data suggest that any disturbances in glucose and likely other carbohydrates can affect this behavioral domain.

Lysine, glutamic acid, and 5-aminovaleric acid positively correlated with the various social behaviors in GEN males vs. AIN males. As mentioned above, 5-aminovaleric acid may weakly inhibit GABAB receptors^68^. Glutamate and GABA neurotransmission have been implicated in mood disorders^70,71^. Mice exposed to subchronic and social defeat stress exhibit elevations in cecal 5-aminovaleric acid and cholic acid^73^. This same study revealed that *Lachnospiraceae* was inversely associated with several cecal metabolites. This bacterium was decreased in GEN males, but no relationships were evident for Lachnospiraceae and the metabolites or behaviors measured.

The limitations of the current study are that we can only establish correlations between the gut microbiota, fecal metabolome, and behavioral changes. To establish causation that bacterial/metabolite changes due to developmental exposure to BPA or GEN cause neurobehavioral alterations, fecal microbial samples from individuals exposed to one of these two chemicals would need to be transferred into recipient germ-free mice that lack resident gut microbiota and have not been exposed to such chemicals. If gut microbiome and metabolome are mediating such effects, the prediction would be that these recipient germ-free mice would develop similar neurobehavioral disruptions as donors exposed to one of these chemicals. To perform such studies, we are in the process of establishing collaborations with those at other Institutes who have a germ-free mouse facility. Additionally, LC–MS/MS analysis yielded the most significant metabolite differences. However, even with several analytic approaches, only a handful of these could be identified. As more libraries and information becomes available, we are hoping to be able to identify more of these unknown metabolites in future studies.

In conclusion, the current studies show that developmental exposure to BPA resulted in dose dependent effects with the LD but not the UD resulting in California mice spending more time engaging in repetitive behaviors, which is considered a type of autistic-like behavior. Similarly, GEN exposed individuals were also more likely to exhibit such behaviors. This group also showed socio-communicative disturbances. BPA and GEN induced sex-dependent differences in gut metabolite profiles with exposed females showing increased number of metabolites involved in carbohydrate metabolism and synthesis. In contrast, males exposed to BPA or GEN had alterations in lysine degradation, phenylalanine and tyrosine metabolism, and urea cycle. Multiomics integrative correlation analyses revealed several correlations between BPA/GEN induced gut microbial, fecal metabolite, and neurobehavioral alterations. It remains to be determined whether such EDC-induced gut microbiota and fecal metabolite change alters neurobehavioral programming through the microbiome-gut-brain axis. If so, reversal of such effects through probiotic or post-biotic supplementation may provide a treatment avenue to mitigate the deleterious effects of BPA and GEN.

## Materials and methods

Materials and methods for collection of fecal samples and isolation of fecal microbial DNA, 16S rRNA sequencing, bioinformatics and amplicon analyses, gut metabolome analyses, further identification of non-targeted metabolites of LC–MS/MS data, and gut metabolome statistical analyses are including in Supplementary Information.

### Animals and treatments

As detailed previously^18–20,74^, founder adult California mouse (*Peromyscus californicus*) males and females were purchased from the *Peromyscus* Genetic Stock Center (PGSC) at the University of South Carolina (Columbia, SC). These animals were 60–90 days of age and free of common rodent pathogens. They were shipped to the University of Missouri and placed in quarantine, along with sentinel mice, for a minimum of 8 weeks to ensure that they did not carry any transmittable diseases. No diseases have been identified to date in any sentinel or colony animals. After the quarantine period, the animals were moved to the Animal Sciences Research Center (ASRC), where we have our own breeding colony established. Additional animals have been routinely purchased from the PGSC, using similar methods, in order to maintain the outbred status of the colony. The breeder pairs used for these studies were descendants of those obtained from the PGSC as we had to ensure that they were on a phytoestrogen-free diet from the time of birth until sexual maturity. All experiments were approved by the University of Missouri Animal Care and Use Committee (Protocol Numbers 8693 and 9590) and performed in accordance with the recommendations in the National Institutes of Health Guide for the Care and Use of Laboratory Animals.

Breeder pairs and those with pups were housed in polysulfone cages (17 inches × 8.25 inches, Allentown, NJ) and weaned F1 offspring were singly housed in polypropylene cages (10.5 inches × 6 inches, Allentown, NJ, USA) to reduce background exposure to bisphenol A (BPA). Polypropylene cages have minimal BPA^75^. New polysulfone cages have detectable amount of BPA, but this does not appear to be the case for older cages^75^, which were used in the current study. We could not use polypropylene cages for the breeder pairs as we have not been able to find large cages made of this material. Our animal facility requires the larger cages for the breeder pairs and those with pups as California mice offspring are dependent upon both maternal and paternal care^17^. Both polypropylene and polysulfone cages are preferred over polycarbonate cages, which contain the greatest amounts of BPA^75^. Glass water bottles were provided. Virgin females (8–12 weeks of age) were randomly assigned to receive one of four diets: (1) a low phytoestrogen AIN 93G diet (CTL diet, TD.95092; Envigo, Madison, WI) supplemented with 7% corn oil by weight to minimize potential phytoestrogenic contamination that would otherwise come from using soy oil as the primary fatty acid source; (2) AIN base diet supplemented with 250 mg/kg feed weight of genistein (GEN, TD.10691); (3) AIN base diet supplemented with a “lower dose- LD” of BPA (5 mg/kg feed weight, TD.170514); or 4) AIN base diet supplemented with a “upper dose-UD” of BPA (50 mg/kg feed weight, TD.09518), which we have documented to lead to internal serum concentrations in rodents^16,76^ approximating those previously measured in pregnant women and the human population in general that is unknowingly exposed to this chemical^77–80^. For Diets 2–4, they were identical to the parental AIN 93G base diet in terms of nutrient composition. BPA and GEN were shipped to Envigo, and their laboratory animal certified nutritionists added the chemicals to the AIN93G base diet. In pilot studies, we have shown that California mice on all four of these diets consume the same amount, suggestive that the added chemicals do not affect their appetence or diet palatability. The P_0_ dams remained on the assigned diet for two weeks prior to mating (periconceptional period), throughout gestation, and throughout lactation, as described previously^15,16,18^. Dams remained on their assigned diet until pups were weaned, as brain development in rodents extends throughout the post-natal period^81,82^. This method was used to replicate the maternal diet exposure of fetuses and neonates to GEN and BPA, which can be transmitted to the offspring both across the placenta and through the milk. Concentrations of BPA or GEN are listed above on a mg/kg feed weight basis as we did not directly measure internal serum concentrations reached in dams or individual pups. This listing is also to be consistent with our previous studies and those of others who have shown exposure to BPA or GEN during this pre- and post-natal period induce later neurobehavioral and epigenetic changes^15,16,18,76,83–87^. However, in previous studies we have measured internal BPA serum concentrations achieved in laboratory mice and deer mice on similar BPA diets^16,76^. Deconvolution analysis based on comprehensive pharmokinetic profiling revealed that female mice consuming the AIN diet supplemented with 100 mg BPA-*d*_6_/kg feed weight reached an internal concentration of 15.6 mg/kg between 0 and 11 h after beginning the dietary exposure^76^. Female deer mice chronically fed the same 50 mg BPA/kg feed weight diet as used herein had mean unconjugated (free) native BPA concentrations at 5.48 ± 2.07 ng/ml with a range of 0.79–19.3 ng/ml^16^. These measurements were performed by Dr. K. Kannan’s laboratory; he is considered a leading authority in measuring circulating BPA concentrations. As detailed above, these internal serum concentrations are comparable to those previously identified in humans, including pregnant women, unknowingly exposed to this chemical^77–80^. Moreover, a recent report suggests that flawed techniques by other groups have likely resulted in under-estimation of actual internal concentrations in humans chronically exposed to this chemical^88^. The GEN concentration tested has previously been shown to affect various parameters in mice^83–85^ and results in similar circulating concentrations as in humans consuming soy-rich diets^89^.

Offspring were weaned at 30 days of age and placed on the AIN (CTL) diet. This weaning age is considered standard for California mice^90,91^, who require more time with both parents relative to mice (*Mus musculus*) who only receive maternal care. During the late post-natal period, it is possible that the pups may directly consume some of the food, but the primary routes of exposure would still be considered gestational and lactational.

From each litter, one male and one female pup were randomly selected for follow-up behavioral, gut microbiome, and metabolome analyses. This approach was done to avoid litter bias effects. The remaining pups were used as strangers in the social behavioral testing detailed below or for other studies. It should be noted that in California mice, the average litter size is ~ 3, as females only has four caudal nipples to nurse the pups^90,91^. For all analyses in the current project, the same male and female offspring were tested for these various studies such that correlation analyses could be performed. The number of replicates included were: 10 AIN males, 10 AIN females, 10 GEN males, 10 GEN females, 10 UD-BPA) males, 10 UD-BPA females, 9 low-dose BPA (LD-BPA) males, and 10 LD-BPA females. Appropriate number of replicates needed for significant data, especially for the microbiome and metabolome portions of the experiment, were determined in advanced by using previous studies in this area, including our own past work^22,30,92^. We performed a Power Analysis with SAS Power and Sample Size application (PSS, SAS analysis, Cary, NC, USA) for additional verification that this number of replicates should be sufficient. To avoid running into potential circadian differences due to testing too many animals on a given day, the pairings were staggered. Additionally, not all paired animals mated and gave birth on the same day. Thus, for the behavioral tests detailed below the number of animals tested at any given time ranged from 2 to 8 California mice, as dependent upon their age. For all tests, this number of animals could be tested within a reasonable time frame. For the fecal microbiome and metabolome analyses, however, all samples were analyzed at the same time to avoid potential confounding batch effects.

### Barnes maze

At 90 days of age, the experimental animals underwent Barnes maze testing designed to assess spatial learning and memory^15,16,18,43^. The maze apparatus consisted of a circular platform with twelve holes evenly spaced around the perimeter. Eleven of the twelve holes remain closed, while the twelfth hole opened into an escape hole box that the animal could enter. The maze was enclosed by walls with four different intramaze visual cues located above holes 3, 6, 9, and 12. Bright light sources were positioned above the maze to serve as a mildly aversive, non-painful stimulus to encourage them to seek out the escape hole.

Animals were randomly assigned an escape hole at the beginning of the testing week; the assigned hole was consistent for the five-day duration of the testing period. Each test day, the animals were placed in the testing room to acclimate 30 min prior to testing. The experimental animal was then placed under a clean polypropylene box in the center of the maze. At the start of the first trial, the box was removed and trial initiated. The trial ended when the animal entered the correct escape hole or after 5 min elapsed and the individual did not enter the escape hole in which case it was gently guided to the hole on the first day of testing. This testing period is standard for the Barnes maze^16,18,43,93^. The behavioral maze platform and escape holes were cleaned with 70% ethanol between different animals, which also removed any odor cues from previously tested animals. After a 30 min inter-trial interval, a second trial was performed for each individual. Testing was repeated for five consecutive days, and all trials were video recorded with a Sony Handycam HDR-CX440 (San Diego, CA, USA). Animals were placed back into their cages after each trial and the maze, escape hole box, and polypropylene cover box were cleaned with 70% ethanol, which also removed potential odor cues. Video data were analyzed by using ANY-maze software version 6.0 (Stoelting, Wood Dale, IL, USA). The program, which uses a three-point tracking system, determines the movement of the mouse within the maze and analyzed various parameters, including latency to find the correct escape hole, number of approaches to the correct hole, total distance traveled, and velocity.

In the above studies, we sought to test all of the animals at the same age. We realize that in so doing some of the California mice females could have been at different stages of the estrous cycle, which could confound the results. However, the variation between animals was not more than two standard deviations, suggestive that the data across females within a group did not diverge significantly. As with laboratory mice, the estrous cycle length can vary across females. Thus, even if we initiated testing of the females at the same stage of the estrous cycle, they could have started to diverge as they were analyzed in the multi-day behavioral tests.

### Social behavior testing

After completion of the Barnes maze, the test offspring were then tested in Crawley’s sociability and preference for social novelty three-chambered test, as described previously by Refs.^20,44^, which was designed to identify potential social deficits^44^. The three-chambered apparatus had two openings, which allowed the animal to move freely between the three chambers. The left and right chamber contained wire mesh cups that held stranger (novel) individuals. Stranger California mice were chosen based on similar age, same sex as the test subjects, same perinatal diet exposure as test subjects, and having different parental lineage relative to the test subject. As detailed above, individuals designated as strangers were other offspring from the generated litters who themselves were not subject to behavioral testing. Each experimental animal was tested for three trials. Prior to the test, the experimental and stranger animals were acclimated into the testing room for 30 min. In the first trial, the test animal was placed into the center chamber and was acclimated to the test apparatus for 5 min with no stranger individuals present. Both stranger individuals were placed in individual wire mesh cups and acclimated to the testing apparatus for 5 min. In trial 2, the experimental animal was placed in the center chamber and one stranger individual was placed in a wire-mesh cup in the left chamber, and the trial lasted 10 min. In trial 3, the experimental animal was placed in the center chamber and the old “stranger” individual was placed in a wire-mesh cup on the left chamber side, and Stranger 2 was placed in a wire-mesh cup on the right side of the chamber with the test lasting 10 min. These times are identical to those used in previous studies^20,44^ and designed to provide the test individual ample time to interact with novel individuals as a measure of social behaviors but to avoid habituation to them that would impact the results. The total time for the testing procedure takes about 35 min. Each trial was video-recorded by using a Logitech Carl Zeiss Tessar HD 1080P (Newark, CA) camera mounted onto a Joby Gorilla Pod Original Tripod (Daymen US Inc., Petaluma, CA). After each trial, all animals were removed and the apparatus was cleaned with a 70% ethanol solution. This cleaning procedure was also done between testing different animals in this behavioral test. Video data were analyzed by using Observer software version 11.5 (Noldus, Leesberg, VA). Behaviors such as rearing, grooming, and nose-to-nose contacts with the stranger mice (in Trials 2 and 3) were determined, along with the location of the mouse within the three-chambered apparatus.

### Vocalizations

Immediately after Trial 3 of the social behavior testing, test individuals were placed in a clean, empty polypropylene cage and transferred into a polypropylene box lined with 2 inches of acoustic foam paneling (Soundproof Cow, Chambersburg, PA), as we have done previously^20,93^. The box contained a small light source (20-W LED puck lights, Intertek, London, UK) and an Avisoft Bioacoustics CM16/CMPA40-5V microphone (Glienicke, Germany) that was 33 cm from the floor of the box. The microphone was connected to National Instruments USB 6351 data collection board, which was plugged into a Dell OptiPlex 7010 (Dell Incorporated, Roundrock, TX). Audio was recorded for 5 min, as we have done previously^20,93^ to provide a good sampling of the vocalizations produced in isolation. Recordings measured include audible calls and USV that were above 20 kHz and out of the hearing range for humans. The polypropylene box was cleaned with 70% ethanol after each test mouse. Data were collected by using code written by Dr. Katrin Schenk (Randolph College) and MATLAB 2015a.Ink version 8.5.0.197613 (R2015a) software (MathWorks, Natick, MA). Vocalizations were separated from background noise and analyzed with MATLAB software designed by Dr. Katrin Schenk. Categories evaluated include number of syllables, syllable duration, syllable median frequency, average syllable power, and power percent above and below 20 kHz, as detailed previously^20,93^.

### EPM

At PND 97, experimental individuals underwent EPM testing to measure anxiety-like, exploratory, and repetitive behaviors, as we have done previously^15,16,18,94^. The apparatus consisted of two open and two closed arms that were connected to each other and then raised off the floor by a stand measuring 100 cm. The two closed arms had walls that enclosed the mouse from its surroundings and the two open arms did not contain such walls. The open and closed arms were perpendicular to each other. At the beginning of the trial, the test animal was placed in the center and tested for a total duration of 5 min, which is standard for EPM testing^15,16,18^. After the completion of the test, the apparatus was disinfected with 70% ethanol, which also removed any previous odor cues. The trials were recorded with a Sony Handycam HDR-CX440 (San Diego, CA) and then analyzed by using the Observer software version 11.5 (Noldus, Leesberg, VA) with behaviors such as frequency and duration of grooming, frequency and duration of rearing, frequency and duration of head dipping, time spent in open and closed arms, time spent in the center, and entries into open and closed arms determined. More entries and time spent in the open arms are indicative of exploratory, non-anxious behavior; whereas, increased entries and time in the closed arms suggest anxiogenic behaviors.

### Statistical analyses for behavioral data

All dependent variables, including social behaviors in the three-chamber testing, such as time spending with Stranger 1 or 2, nose-to-nose interaction frequency and interaction duration in Trials 2 and 3, USVs, such as total number of calls and power of calls, Barnes maze, such as latency to find correct escape hole, time in the correct zone, and travel speed, and EPM data, such as time spent in open/closed arms, head dipping and rearing, were analyzed as a split plot in space and time, as detailed in our previous studies^20,94^, and with SAS version 9.4 program (SAS Institute, Cary, NC). This approach was done to control for potential litter effects, as one male and one female offspring from each litter were tested. Sources of variation considered included perinatal treatment, offspring sex, and interaction between perinatal treatment and sex. Perinatal treatment effects were determined with dam as the experimental unit and the error term to control further for potential litter effects.

Barnes data were additionally analyzed by using a repeated measurement design with PROC GLIMMIX. As initial analysis indicated a significant three-way interaction between perinatal treatment × sex × day, a cumulative logit analysis for each day was performed, where perinatal treatment, sex and perinatal treatment × sex interaction were modeled.

To examine for differences in social interactions with Stranger 1 between Trials 2 and 3, and differences between Stranger 1 and 2 in Trial 3, behavioral data were analyzed based on perinatal treatment, sex, trial, Stranger 1 or 2, and interactions of all these factors as potential sources of variance.

Differences between perinatal treatment groups and control were determined by Fisher’s protected least-significant difference (LSD). The LSD was only calculated if the overall F test was significant^95,96^. A p value of  ≤ 0.05 was considered significant. All data are presented as actual means. The error bars for all figures and reported data represent the standard error of the mean (SEM).

## Supplementary information

Supplementary file1 (XLSX 389 kb)

Supplementary file2 (XLSX 257 kb)

Supplementary file3 (XLSX 1829 kb)

Supplementary file4 (DOCX 11884 kb)

## Data Availability

All data generated from this current study are contained within the manuscript or as supplementary material. All of the raw gut microbiota data are available on NCBI (BioProject number PRJNA577929, https://dataview.ncbi.nlm.nih.gov/object/PRJNA577929?reviewer=o9j3aooh017qu12rr7r0h2s9n8). All of the raw metabolome data are available at the NIH Common Fund's National Metabolomics Data Repository (NMDR) website, the Metabolomics Workbench: current project ID is PR000932, and DOI number is 10.21228/M8710D. The weblink for the data is https://dev.metabolomicsworkbench.org:22222/data/DRCCMetadata.php?Mode=Project&ProjectID=PR000932&access=LduV2970.
