# Author Correction: Developmental exposure of California mice to endocrine disrupting chemicals and potential effects on the microbiome-gut-brain axis at adulthood

**DOI:** 10.1038/s41598-020-69601-y

**Published:** 2020-07-22

**Authors:** Sarabjit Kaur, Saurav J. Sarma, Brittney L. Marshall, Yang Liu, Jessica A. Kinkade, Madison M. Bellamy, Jiude Mao, William G. Helferich, A. Katrin Schenk, Nathan J. Bivens, Zhentian Lei, Lloyd W. Sumner, John A. Bowden, Jeremy P. Koelmel, Trupti Joshi, Cheryl S. Rosenfeld

**Affiliations:** 1 Christopher S Bond Life Sciences Center, University of Missouri, Columbia, MO 65211 USA; 2 Biomedical Sciences, University of Missouri, Columbia, MO 65211 USA; 3 MU Metabolomics Center, University of Missouri, Columbia, MO 65211 USA; 4 MU Institute of Data Science and Informatics, University of Missouri, Columbia, MO 65211 USA; 5 Food Science and Human Nutrition, University of Illinois, Urbana, IL 61801 USA; 6 Physics, Randolph College, Lynchburg, VA 24503 USA; 7 DNA Core Facility, University of Missouri, Columbia, MO 65211 USA; 8 Department of Biochemistry, University of Missouri, Columbia, MO 65211 USA; 9 Department of Physiological Sciences, College of Veterinary Medicine, University of Florida, Gainesville, FL 32610 USA; 10 Center for Environmental and Human Toxicology, College of Veterinary Medicine, University of Florida, Gainesville, FL 32610 USA; 11 Environmental Health Sciences, Yale University, New Haven, CT 06510 USA; 12 Department of Health Management and Informatics, School of Medicine, University of Missouri, Columbia, MO 65211 USA; 13 Thompson Center for Autism and Neurobehavioral Disorders, University of Missouri, Columbia, MO 65211 USA; 14 Genetics Area Program, University of Missouri, Columbia, MO 65211 USA

Correction to: *Scientific Reports* https://doi.org/10.1038/s41598-020-67709-9, published online 02 July 2020

In this Article, the weblink for readers to access the raw metabolomics data is incorrect.

As a result, within the Results section under the subheading ‘Metabolome’,

“All of the raw metabolome data are available at the NIH Common Fund's National Metabolomics Data Repository (NMDR) website, the Metabolomics Workbench: current project ID is PR000932, and DOI number is 10.21228/M8710D. The weblink for the data is https://dev.metabolomicsworkbench.org:22222/data/DRCCMetadata.php?Mode=Project&ProjectID=PR000932&access=LduV2970.”

should read:

“All of the raw metabolome data are available at the NIH Common Fund's National Metabolomics Data Repository (NMDR) website, the Metabolomics Workbench: current project ID is PR000932. The data can be accessed directly via it's Project DOI: (10.21228/M9710D). This work is supported by NIH grant U2C-DK119886.”.

Also, within the Data Availability section:

“All of the raw metabolome data are available at the NIH Common Fund's National Metabolomics Data Repository (NMDR) website, the Metabolomics Workbench: current project ID is PR000932, and DOI number is 10.21228/M8710D. The weblink for the data is https://dev.metabolomicsworkbench.org:22222/data/DRCCMetadata.php?Mode=Project&ProjectID=PR000932&access=LduV2970.”

should read:

“All of the raw metabolome data are available at the NIH Common Fund's National Metabolomics Data Repository (NMDR) website, the Metabolomics Workbench: current project ID is PR000932. The data can be accessed directly via it's Project DOI: (10.21228/M9710D). This work is supported by NIH grant U2C-DK119886.”.

