# Supplementary material for: Developmental exposure of California mice to endocrine disrupting chemicals and potential effects on the microbiome-gut-brain axis at adulthood
Source: Sci Rep. 2020 Jul 2;10:10902. doi: 10.1038/s41598-020-67709-9 (PMC7331640; doi:10.1038/s41598-020-67709-9)
Supplement: Supplementary file 4 — Supplementary file4 (DOCX 11884 kb) [file 41598_2020_67709_MOESM4_ESM.docx]

**Supplementary Information**

**Developmental Exposure of California Mice to Endocrine Disrupting Chemicals and Effects on the Microbiome-Gut-Brain Axis at Adulthood**

Sarabjit Kaur^1,2^, Saurav J. Sarma^1,3^, Brittney L. Marshall^1,2^, Yang Liu^1,4^, Jessica A. Kinkade^1,2^, Madison M. Bellamy^1,2^, Jiude Mao^1,2^, William G. Helferich^5^, A. Katrin Schenk^6^,

Nathan J. Bivens^7^, Zhentian Lei^1,3,8^, Lloyd W. Sumner^1,3,8^, John A. Bowden^9,10^,

Jeremy P. Koelmel^11^, Trupti Joshi^1,4,12^, Cheryl S. Rosenfeld^1,2,4,13,14^

**Affiliations:**

^1^Christopher S Bond Life Sciences Center, University of Missouri, Columbia, MO 65211 USA

^2^Biomedical Sciences, University of Missouri, Columbia, MO 65211 USA

^3^MU Metabolomics Center, University of Missouri, Columbia, MO 65211 USA

^4^Informatics Institute, University of Missouri, Columbia, MO 65211 USA

^5^Food Science and Human Nutrition, University of Illinois, Urbana, IL 61801 USA

^6^Physics, Randolph College, Lynchburg, VA 24503 USA

^7^DNA Core Facility, University of Missouri, Columbia, MO 65211 USA

^8^Department of Biochemistry, University of Missouri, Columbia, MO 65211 USA

^9^Department of Physiological Sciences, University of Florida, College of Veterinary Medicine, Gainesville, FL 32610 USA

^10^Center for Environmental and Human Toxicology, University of Florida, College of Veterinary Medicine, Gainesville, FL 32610 USA

^11^Environmental Health Sciences, Yale University, New Haven, CT 06510 USA

^12^Department of Health Management and Informatics, School of Medicine, University of Missouri, Columbia, MO 65211 USA

^13^Thompson Center for Autism and Neurobehavioral Disorders, University of Missouri, Columbia, MO 65211 USA

^14^Genetics Area Program, University of Missouri, Columbia, MO 65211 USA

**Short Title: Xenoestrogens and Microbiota-Brain Effects**

**Keywords:** Phytoestrogens; Xenoestrogens; Intestinal bacteria; Diet; Rodent models; Bioinformatics; Autism; ASD; Metabolome; Developmental; Prenatal; Multi-omics

**Correspondence:** [rosenfeldc@missouri.edu](mailto:rosenfeldc@missouri.edu)

**Supplementary Material**

*Collection of fecal samples and isolation of fecal microbial DNA*

After the suite of behavioral tests were completed, each animal was placed in a cage alone without any bedding. Four to five fecal boli were collected from each animal and placed in sterile 2 mL cryogenic vials (Corning Incorporated, Corning, NY) and placed in liquid N_2_ until they were transferred to a -80℃ freezer, whereupon they were stored until being used for gut microbiota or metabolome analysis. The fecal microbial DNA was isolated from a portion of the fecal boli collected using the Invitrogen PureLink Microbiome DNA Purification Kit (Thermofisher Scientific, Waltham, MA) and in accordance with the manufacturer’s protocol. The quantity of DNA isolated was measured using Qubit 3.0 Fluorometer (Life Technologies, Grand Island, NY). The number of replicates (n = 9 to 10 per group) tested is comparable to other studies examining how *in utero* environmental changes can affect gut bacterial populations and have shown that such sample sizes can result in statistical differences between offspring groups ^1,2^.

*16S rRNA sequencing*

The University of Missouri (MU) DNA Core Facility prepared bacterial 16S ribosomal DNA amplicons from extracted fecal DNA by amplification of the V4 hypervariable region of the 16S rDNA with universal primers (U515F/806R) flanked by Illumina standard adapter sequences ^3,4^. The rest of the procedures were performed as described previously ^5,6^. The resulting amplicon pool was evaluated using the Advanced Analytical Fragment Analyzer automated electrophoresis system, quantified with a Qubit fluorometer using a quant-iT HS dsDNA reagent kit (Invitrogen), and diluted according to Illumina’s standard protocol for sequencing on the MiSeq.

*Bioinformatics and amplicon analyses*

Paired-end Illumina MiSeq DNA reads were joined using join_paired_ends.py and combined using add_qiime_labels.py from QIIME 1.9.1 ^7^. Uclust ^8^ was used to clean contigs and remove those with E > 0.5, (<http://drive5.com/usearch/manual/exp_errs.html>). Contigs were clustered to 97% identity against DNA sequences in the Greengenes database ^9^, version 13_8, using the QIIME ^10^, version 1.9.1, script pick_open_reference_otus.py, which obviates chimera and PCR error detection with all reads clustered. After OTU selection, we filtered out OTUs with less than ten observation counts using the script filter_otus_from_otu_table.py. For alpha-diversity in F_1_ samples, Chao1 (species richness), Shannon (species diversity) values and rarefaction matrices were calculated and plotted using the alpha_rarefaction.py script in the QIIME package ^10^. Measurements of beta-diversity were facilitated by the QIIME script beta_diversity.py and jackknifed_beta_diversity.py with PCoA plots generated. Visualization of taxonomy bar-charts were generated using summarize_taxa_through_plots.py from QIIME packages. Permutational multivariate analysis of variance (PERMANOVA) was used to examine for significant differences between groups.

For the subsequent differential abundant OTU and taxonomy analysis, we used DESeq2 ^11^ and negative binomial Wald test using scripts differential_abundance.py as implemented in QIIME. We selected significant OTUs based on an adjusted p-value at the 0.05 level mapping to the Greengenes database. Regulation was determined by assigned log2 fold change calculated from differential analysis. With these methods, relevant pairwise comparisons were performed.

*Gut metabolome analyses*

To 10 mg of each fecal sample, 1.0 mL of 80% methanol containing 18 µg/mL of umbelliferone was added, followed by sonication and vortex for 20 s each. Samples were shaken in an orbital shaker for 2 hours at 140 rpm and then centrifuged at 3000g for 40 min. 0.5 mL of the supernatant was transferred to an autosampler vial for liquid chromatography with tandem mass spectrometry (LC-MS/MS) analysis. LCMS analysis was performed on a Bruker maXis impact II quadrupole-time-of-flight mass spectrometer coupled to a Waters ACQUITY UPLC system. Separation was achieved on a Waters C18 column (2.1 x 150 mm, BEH C18 column with 1.7-um particle size) using a linear gradient and mobile phase A (water with 0.1% formic acid) and B (acetonitrile). Solvent B was increased from 5% to 70% over 30 min, then to 95% over 3 min, held at 95% for 3 min, then returned to 5% for equilibrium. The flow rate was 0.56 mL/min and the column temperature was 60 ^°^C. Mass spectrometry was performed in the positive electrospray ionization mode with the nebulization gas pressure at 43.5 psi, dry gas of 12 L/min, dry temperature of 250 ^°^C and a capillary voltage of 4000V. Mass spectral data were collected from 100 and 1500 *m/z* and were auto-calibrated using sodium formate after data acquisition. Data were extracted and normalized using Bruker’s Metaboscape 4.0 software followed by statistical analysis using MetaboAnalyst 4.0 software ^12,13^.

Following extraction for LCMS analysis, 1.5 mL of CHCl_3_ containing 10 µl/mL of docosanol was added to each of the samples, sonicated and vortexed for 20 s each and incubated at 50 ^°^C for 1 hour. 1 mL of HPLC grade water containing 25 µg/mL of ribitol was added to each sample, sonicated and vortexed for 20 s each and incubated for 1 hour at 50 ^°^C. Samples were centrifuged at 3000g for 40 min and allowed to equilibrate for 5 min for separation of layers. For each sample, 1 mL of supernatant was removed from the upper layer and transferred to an autosampler vial for polar gas chromatography–mass spectrometry (GC-MS) analysis. Syringes were washed with chloroform and transferred 1 mL solution from the bottom layer of the supernatant to another auto sampler vial for non-polar GC-MS analysis. One pooled sample was prepared for polar and non-polar GC-MS by combining a 10 µL aliquot of solution drawn from each sample in the respective batch. All of the extracted sample solutions were dried in the autosampler vials using a ultra-high purity nitrogen stream.

Dried samples for polar GC-MS analysis were methoximated in pyridine with 50 μL of 15 mg/mL methoxyamine hydrochloride, and then trimethylsilylated with 50 μL MSTFA (N-methyl-N-(trimethyl-silyl) trifluoroacetamide) + 1% TMCS (chlorotrimethylsilane) reagent ^14^. Samples for non-polar GC-MS analysis were reconstituted in 50 µL of pyridine followed by trimethylsilylation with 50 µL of MSTFA + 1% TMCS. The derivatized extracts were then analyzed for non-targeted metabolic profiling using an Agilent 6890 GC coupled to a 5973N MSD mass spectrometer with a scan range from *m/z* 50 to 650 (Agilent Technologies, Inc., Santa Clara, CA). 1 µL of sample was injected into the GC column with a split ratio of 1:5 for polar GC-MS and 1:1 for non-polar GC-MS analysis. Separation was achieved with a temperature program of 80 ^°^C for 2 min, then ramped at 5 ^°^C /min to 315 ^°^C and held at 315 ^°^C for 12 min, a 60 m DB-5MS column (J&W Scientific, 0.25 mm ID, 0.25 μm film thickness) and a constant flow of 1.0 mL/min of helium gas. A standard alkane mix was used for GC-MS quality control and retention index calculations. The data from the pooled sample were deconvoluted using AMDIS and annotated through mass spectral and retention index matching to an in-house constructed spectra library. The unidentified components were then searched and identified using spectral matching to a commercial NIST17 mass spectral library. The combined identifications were saved as an .ELU file, and the abundance of the ions in all the other samples were extracted using custom MET-IDEA software. The abundances were then normalized to the internal standard, ribitol, and the normalized values were used for statistical comparisons using Metaboanalyst 4.0 software.

*Further identification of non-targeted metabolites of LC-MS/MS data*

Non-targeted identification of metabolites in positive ion polarity was performed using MS-DIAL version 3.82 (<http://prime.psc.riken.jp/>) ^15^ and the included public MS/MS libraries. Data-dependent MS/MS from all sample groups was searched against. The following MS-DIAL parameters were used: MS-DIAL version 3.66 was used by the following parameters: accurate mass tolerance (MS^1^) tolerance, 0.01 Da; MS^2^ tolerance, 0.025 Da; maximum charge number, 2; smoothing method, linear weighted moving average; smoothing level, 3; minimum peak width, 5 scans; minimum peak height, 1000 in positive ion mode; mass slice width, 0.1 Da; sigma window value, 0.5; MS^2^Dec amplitude cut off, 0; exclude after precursor, true; keep isotope until, 0.5 Da; keep original precursor isotopes, false; exclude after precursor, true; MS^1^ for identification, 0.5 Da; accurate mass tolerance (MS^2^) for identification, 0.5 Da; identification score cut off, 70%; relative abundance cut off, 0; top candidate report, true; retention time tolerance for alignment, 0.1 min; MS1 tolerance for alignment, 0.015 Da; peak count filter, 0; adduct ion setting, [M+H]^+^, [M+Na]^+^, and [M+ACN+H]^+^. Note that 0.5 Da was used for the MS^1^ and MS^2^ identification tolerance in order to include library hits from libraries which were developed without high resolution. The data were run separately with a tolerance of 0.01 Da in order to determine MS^2^ identifications matching with high mass accuracy/resolution. Finally, the resulting annotations were manually interrogated using the MS-DIAL interface, and MS^2^ matches where only the precursors were determined without any fragment matches were flagged as low confident hits.

*Gut metabolome statistical analyses*

Multivariate statistical analyses including 2D principal component analysis (PCA), partial least squares-discriminant analysis (PLS-DA), analysis of variance (ANOVA), T-test, box plots, and volcano plots were performed with the MetaboAnalyst 4.0 program after data pre-treatments, *i.e.,* normalization to the sum, log transformation and Pareto scaling (<http://www.metaboanalyst.ca/>). Changes in metabolite abundances were considered statistically significant when their *P* values were ≤ 0.05. This program was also used to determine the overall metabolite changes in the CON (AIN) vs GEN, CON vs HD-BPA, CON vs LD-BPA, GEN vs UD-BPA, GEN vs LD-BPBA and UD-BPBA vs ULD-BPA groups in both male and female mice.

*Integrative correlation analyses*

We used the mixOmics R package ^16^ to correlate the bacterial genera changes simultaneously with fecal metabolome and behavioral results, which enabled the integration of the microbiome, metabolome, and mouse behavioral data, including Barnes, EPM, social testing, and vocalization results. We conducted sparse discriminant analysis with partial least square regression with function ‘block.splsda’. The circos plot was generated by using the ‘circosPlot’ function with correlations calculated using the method from González, et al. ^17^ and 0.7 correlation was used as the cutoff.

**Supplementary Figures**

**
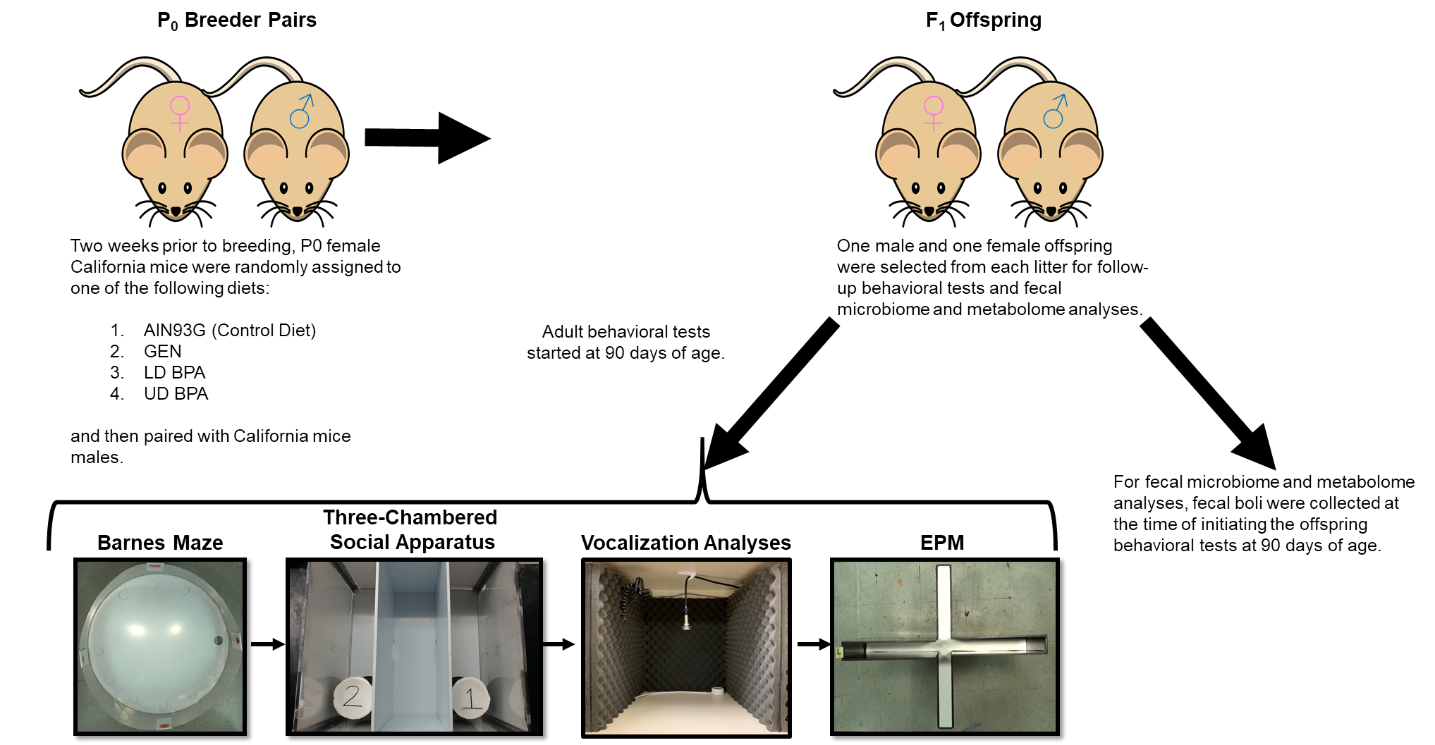
**

**Supplementary Figure 1.** General experimental design to test the effects of developmental exposure to GEN, LD BPA, and UD BPA on adult male and female California mice behaviors and fecal metabolome and microbiome. Adult behavioral tests were conducted starting at 90 days of age in the order listed that also then tests the animals in the least to the potentially most anxiety-inducing tests. Fecal boli were collected at the initiation of the behavioral tests to examine the fecal microbiome and metabolome profiles in the different groups.


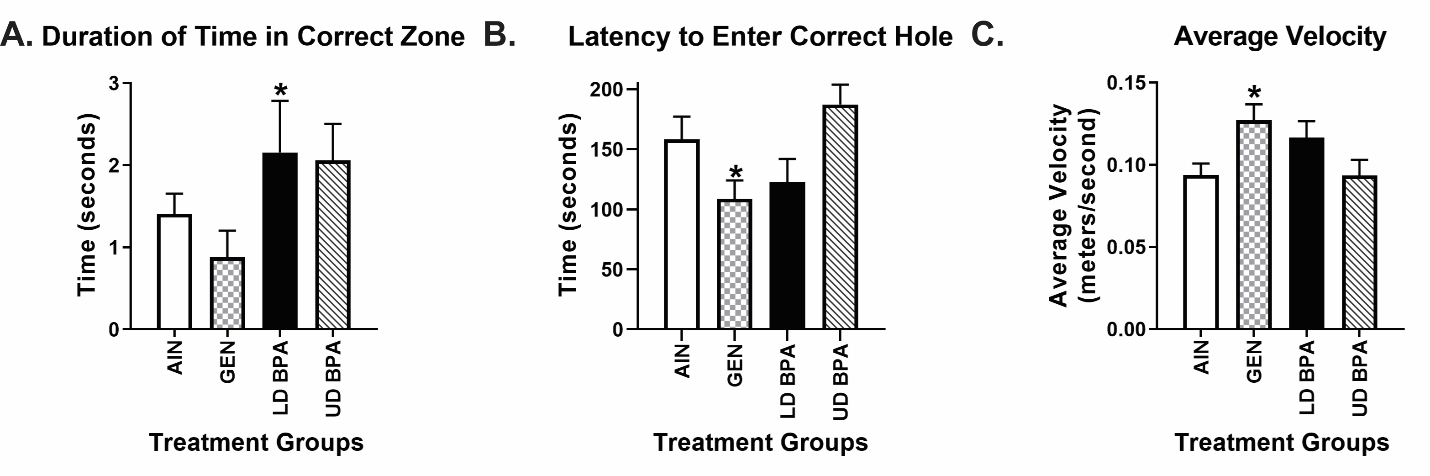


**Supplementary Figure 2.** Barnes maze results. A) Duration of time spent in correct zone. LD BPA spent more time in the correct zone, but no other differences were observed. *p= 0.02. B) Latency to enter the correct hole. GEN exposed individuals exhibited shorter latency to enter the correct hole vs. AIN controls. *p = 0.01. C) Average velocity in the Barnes maze. GEN exposed individuals were speedier in the Barnes maze relative to AIN controls. *p=0.002.

**
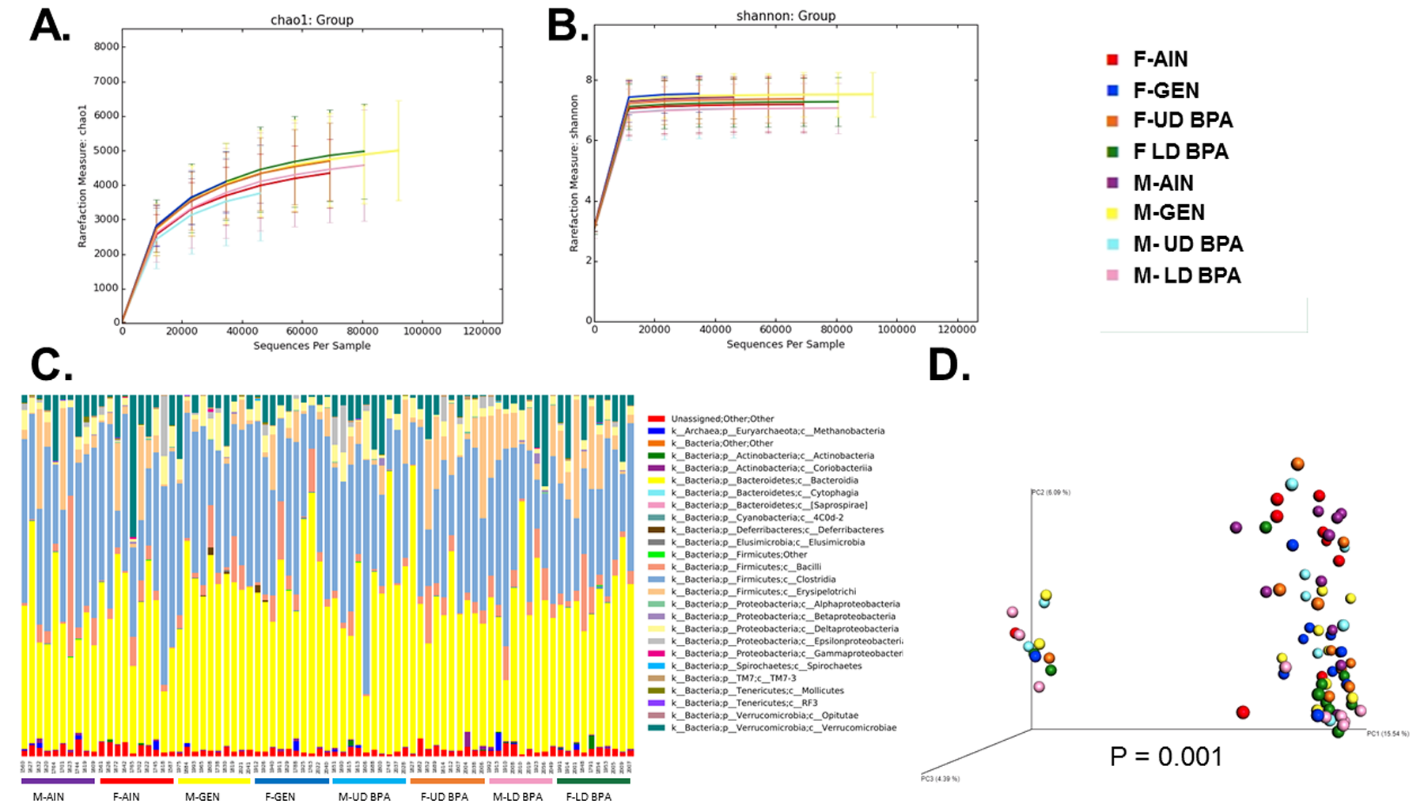
**

**Supplementary Figure 3.** Measures of α- and β-diversity in all groups. A and B) Developmental exposure of males and females to GEN did not affect overall differences in species richness (as shown by Chao1 results) and diversity (as shown by Shannon results). The data were plotted using phyloSeq R package plot_richness function ^18^. C) Bar plot analysis of the most abundant bacterial species in all treatment groups for F1 males and females. D) 3D PCoA analysis F1 male and female offspring results. Weighted PERMANOVA (p = 0.001).

**
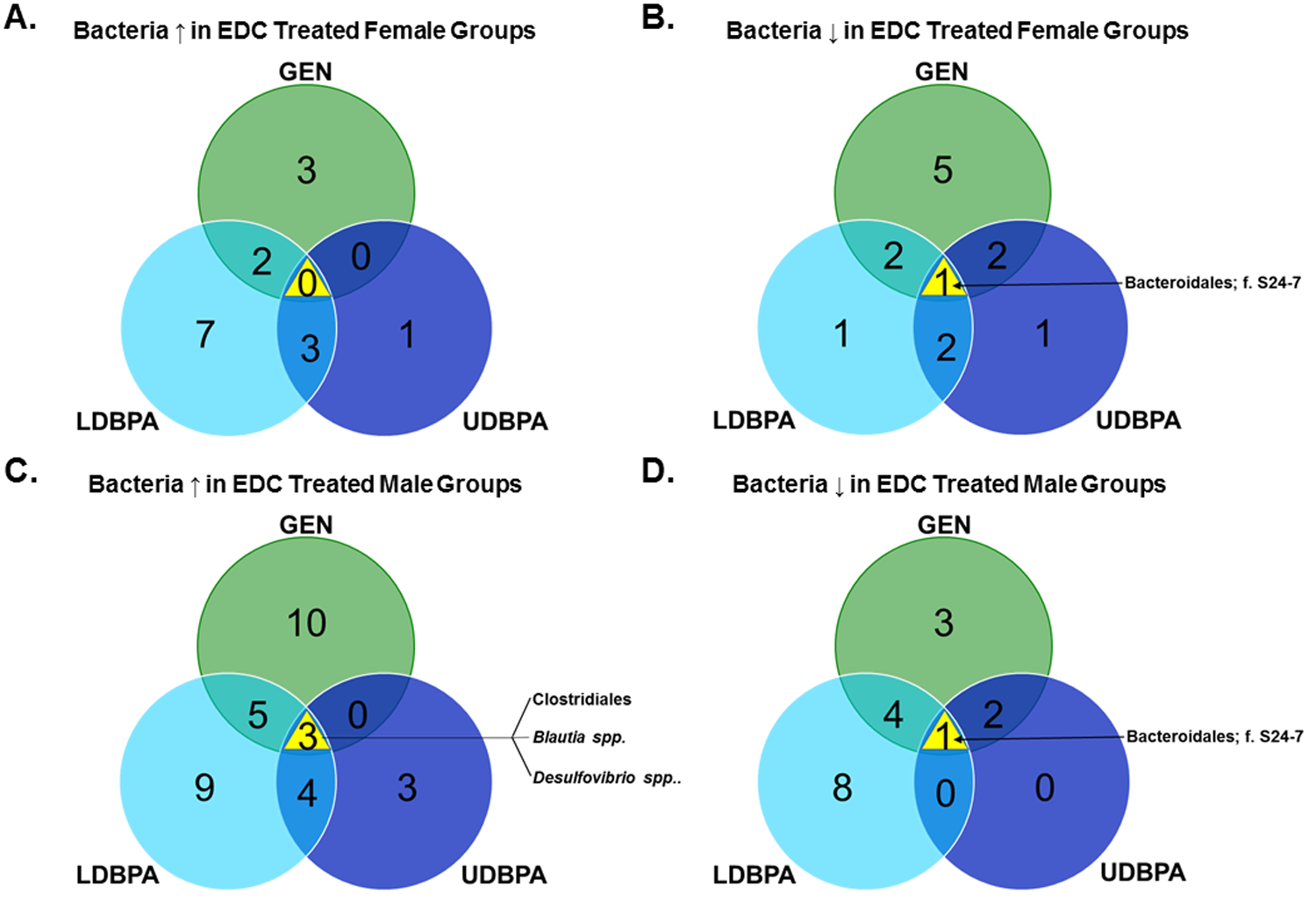
**

**Supplementary Figure 4.** Venn diagram comparisons for bacterial differences in EDC treated groups. A) Bacteria greater in EDC exposed female groups. B) Bacteria decreased in EDC exposed female groups. C) Bacteria greater in EDC exposed male groups. D) Bacteria decreased in EDC exposed male groups.

**
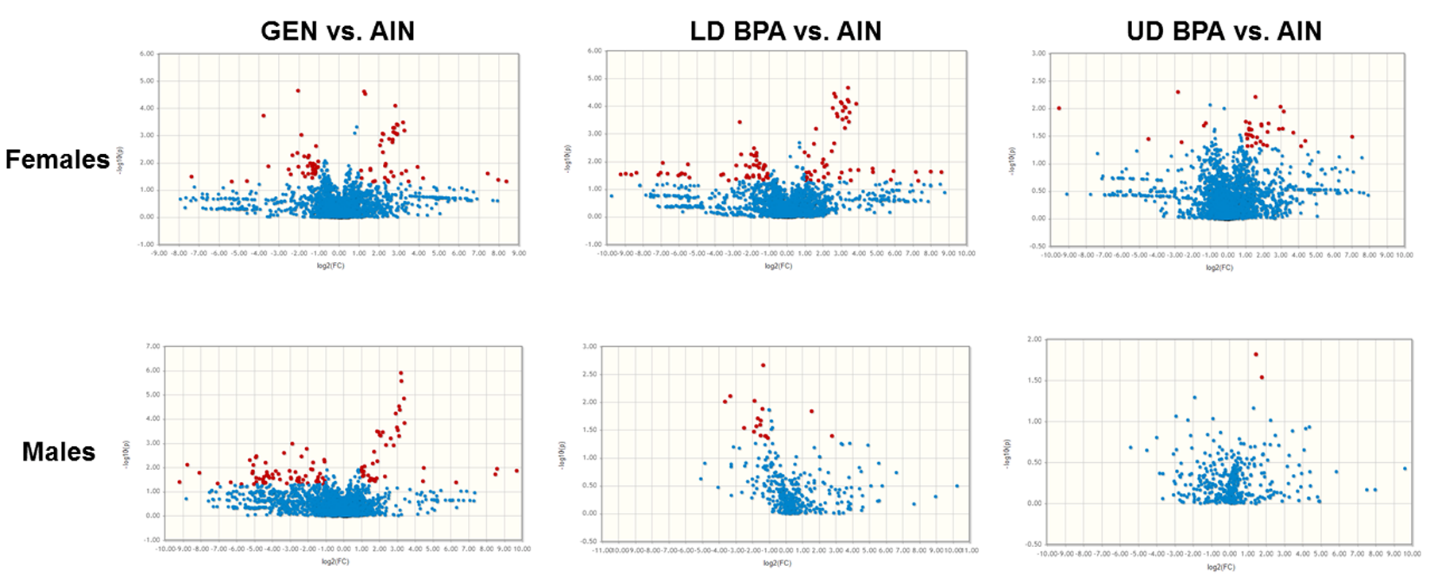
**

**Supplementary Figure 5.** Volcano plots for GC-MS polar fraction results. Y axis represents the negative value of log of *p* value. The significant threshold was 1.301, ie, -log_10_ (0.05). The x axis is the log base 2 of fold change (FC). A value of 1, ie, log_2_(2), corresponding to a 2-fold change was used as a cutoff for increase or decrease. Blue dots represent non-significantly different metabolites (P>0.05), red dots represent significantly different metabolites (p<0.05). GEN: Genistein; AIN: Control; LD BPA: lower dose bisphenol A; UD BPA: upper dose bisphenol A.

**
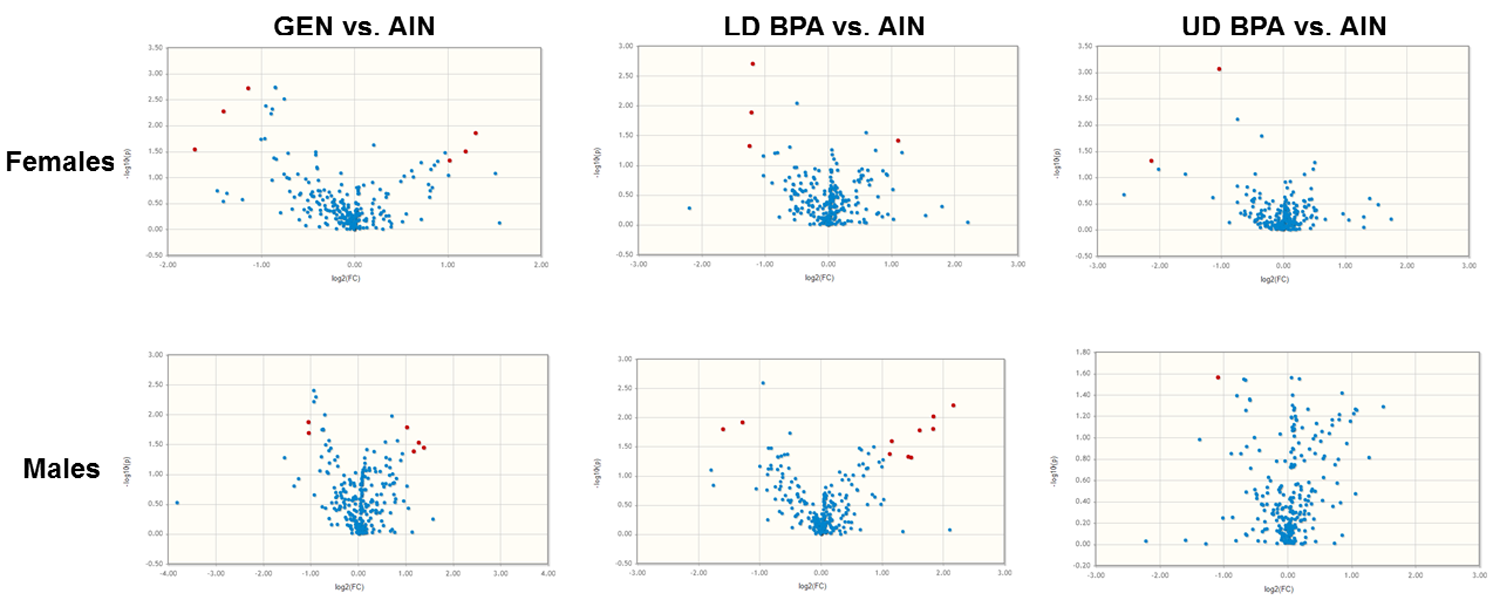
**

**Supplementary Figure 6.** Volcano plots for GC-MS non-polar fraction results. Y axis represents the negative value of log of *p* value. The significant threshold was 1.301, ie, -log_10_ (0.05). The x axis is the log base 2 of fold change (FC). A value of 1, ie, log_2_(2), corresponding to a 2-fold change was used as a cutoff for increase or decrease. Blue dots represent non-significantly different metabolites (P>0.05), red dots represent significantly different metabolites (p<0.05). GEN: Genistein; AIN: Control; LD BPA: lower dose bisphenol A; UD BPA: upper dose bisphenol A.

**
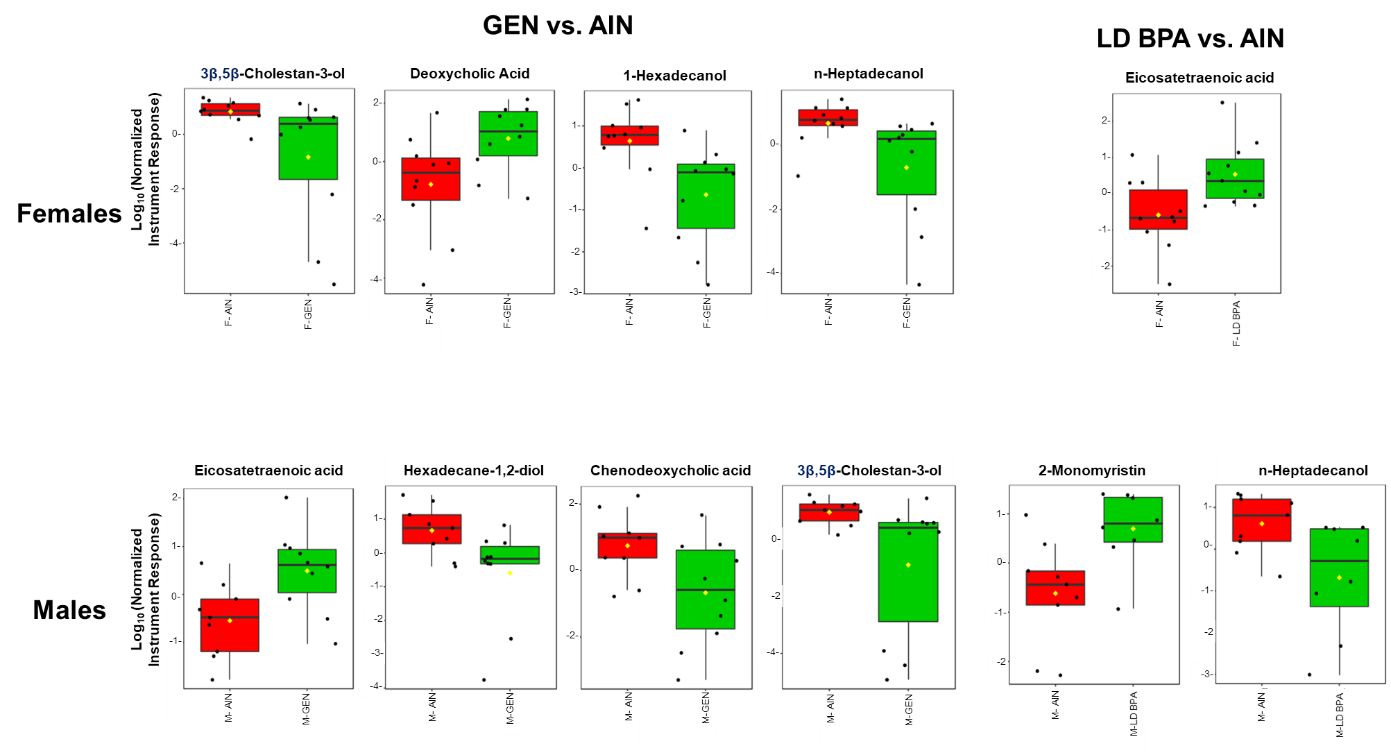
Supplementary Figure 7.** GC-MS non-polar fraction results for female and male groups. Results for this comparison are shown with box and whisker plots. The median is designated with the vertical line inside the box. The yellow dot indicates the mean value. Lines outside indicate the lowest and highest observations. Each replicate is shown as a single black dot. Graphs were generated with the MetaboAnalyst Program v. 4.0 (<https://www.metaboanalyst.ca/>).

**
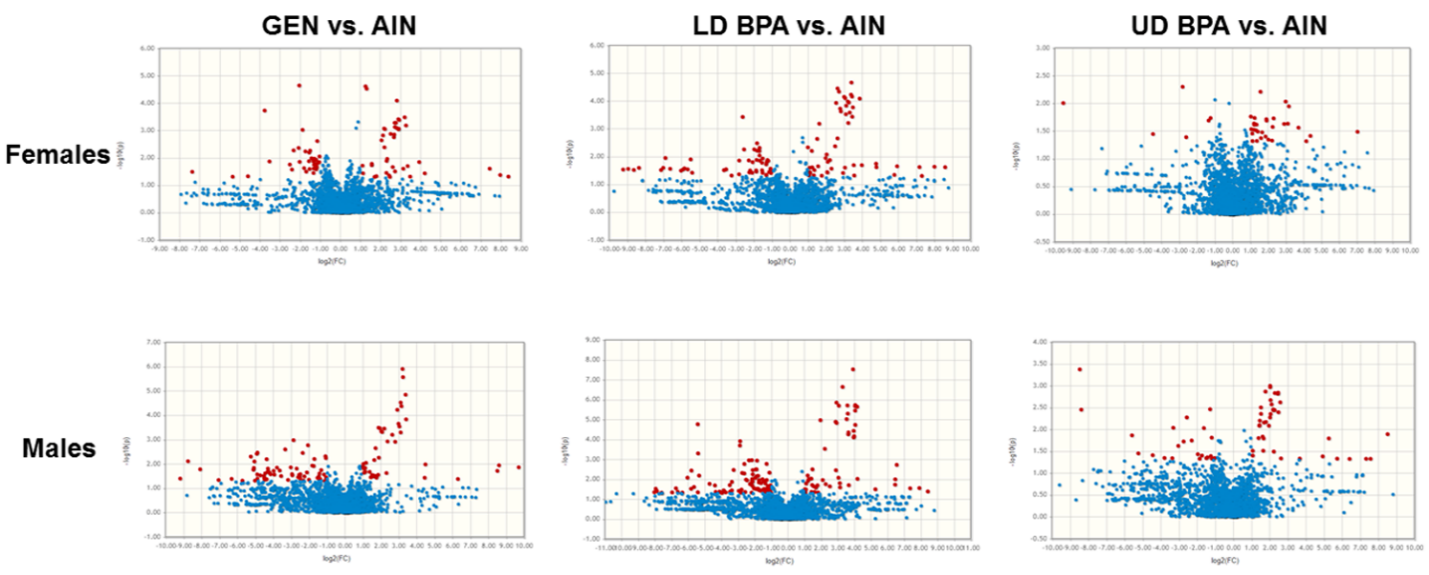
**

**Supplementary Figure 8.** Volcano plots for LC-MS/MS results. Y axis represents the negative value of log of *p* value. The significant threshold was 1.301, ie, -log_10_ (0.05). The x axis is the log base 2 of fold change (FC). A value of 1, ie, log_2_(2), corresponding to a 2-fold change was used as a cutoff for increase or decrease. Blue dots represent non-significantly different metabolites (P>0.05), red dots represent significantly different metabolites (p<0.05). GEN: Genistein; AIN: Control; LD BPA: lower dose bisphenol A; UD BPA: upper dose bisphenol A.


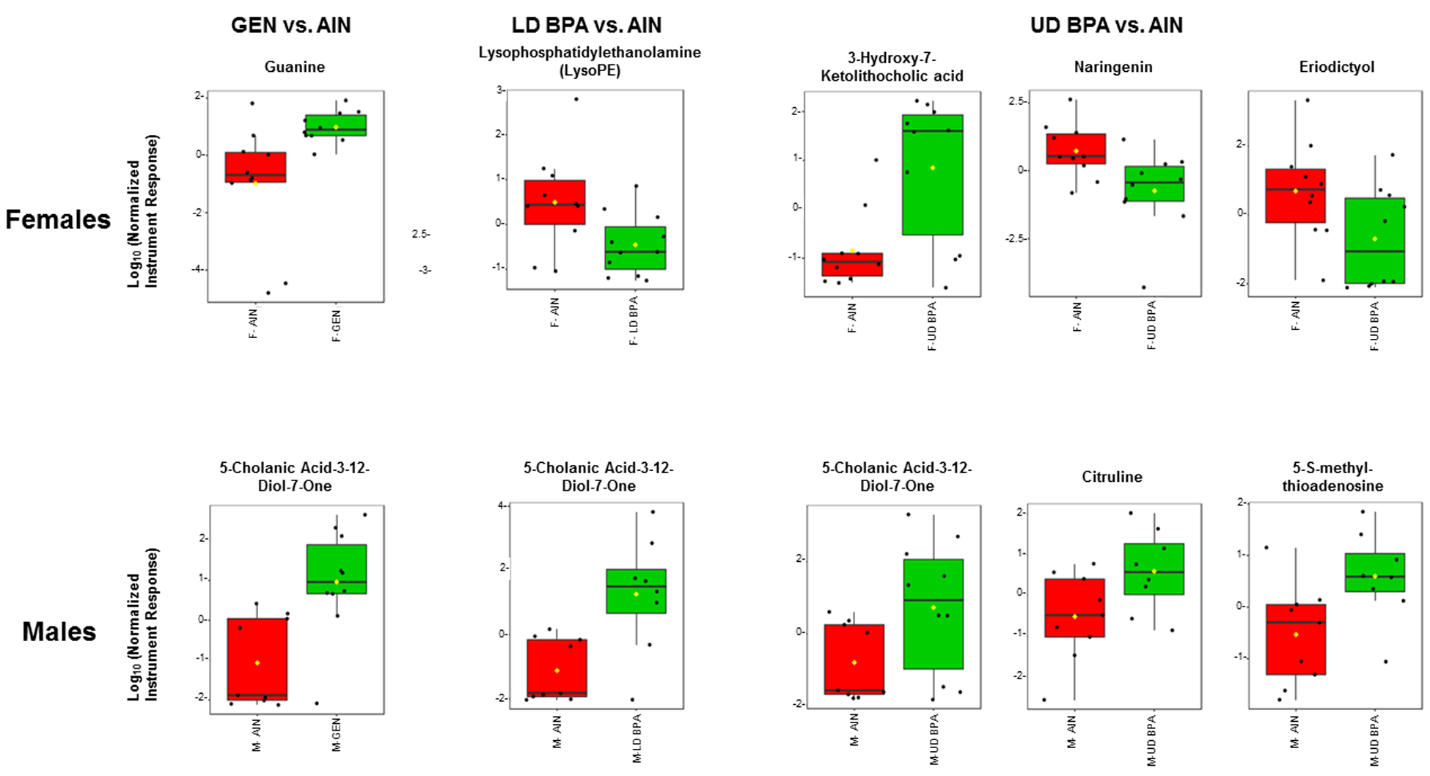


**Supplementary Figure 9.** LC-MS/MS results for female and male groups. Results for this comparison are shown with box and whisker plots. The median is designated with the vertical line inside the box. The yellow dot indicates the mean value. Lines outside indicate the lowest and highest observations. Each replicate is shown as a single black dot. Graphs were generated with the MetaboAnalyst Program v. 4.0 (<https://www.metaboanalyst.ca/>).

**
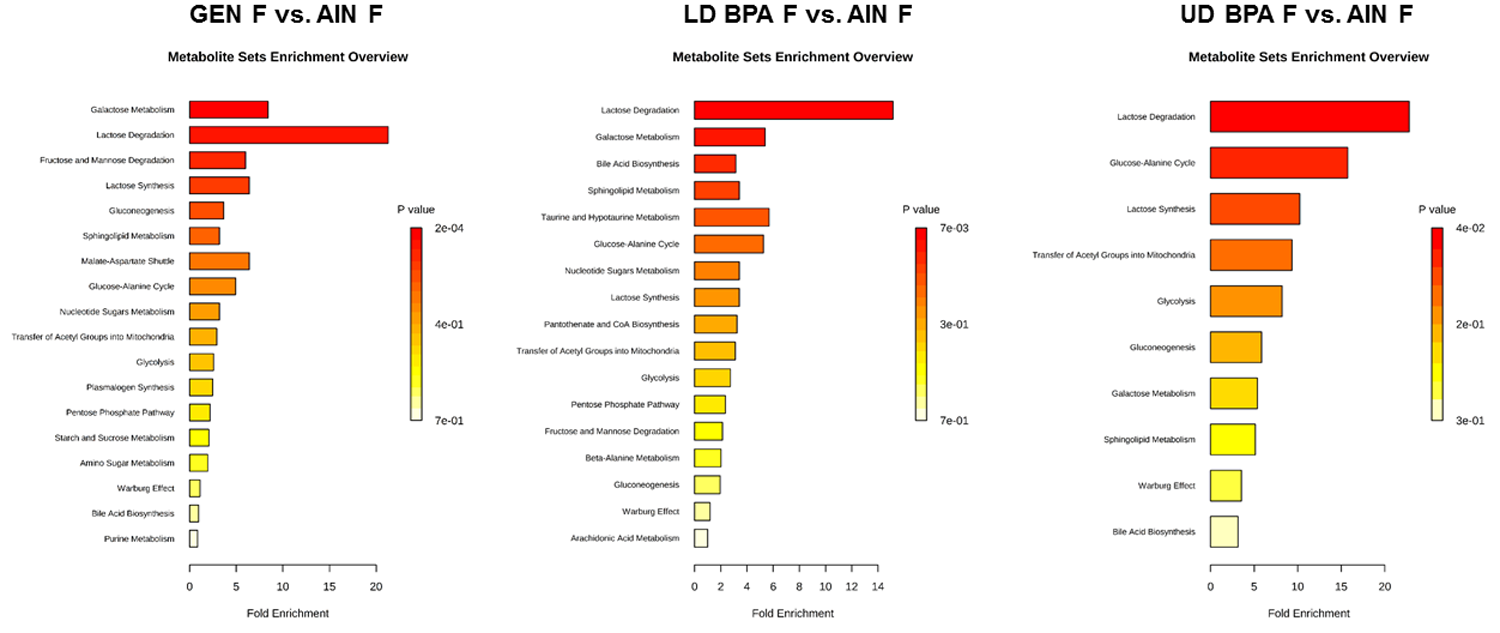
**

**Supplementary Figure 10.** Enrichment analysis for metabolite differences in female groups. Enrichment analyst was performed with MetaboAnalyst Program v. 4.0 (<https://www.metaboanalyst.ca/>) to determine which categories are altered in females developmentally exposed to GEN, LD BPA, and UD BPA.

**
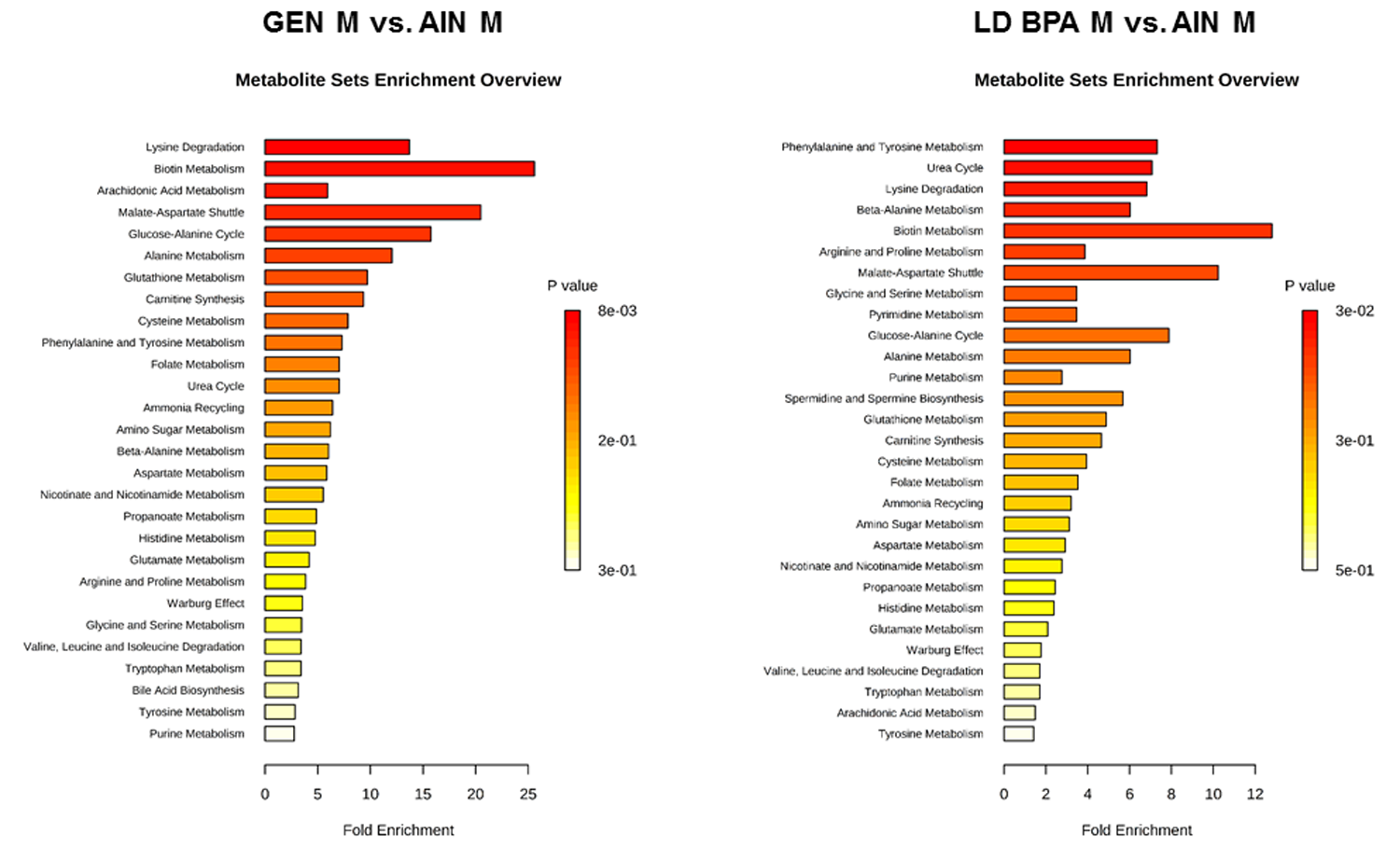
**

**Supplementary Figure 11.** Enrichment analysis for metabolite differences in male groups. These were done as detailed in **Supplementary Figure 10** for the three male treated groups.


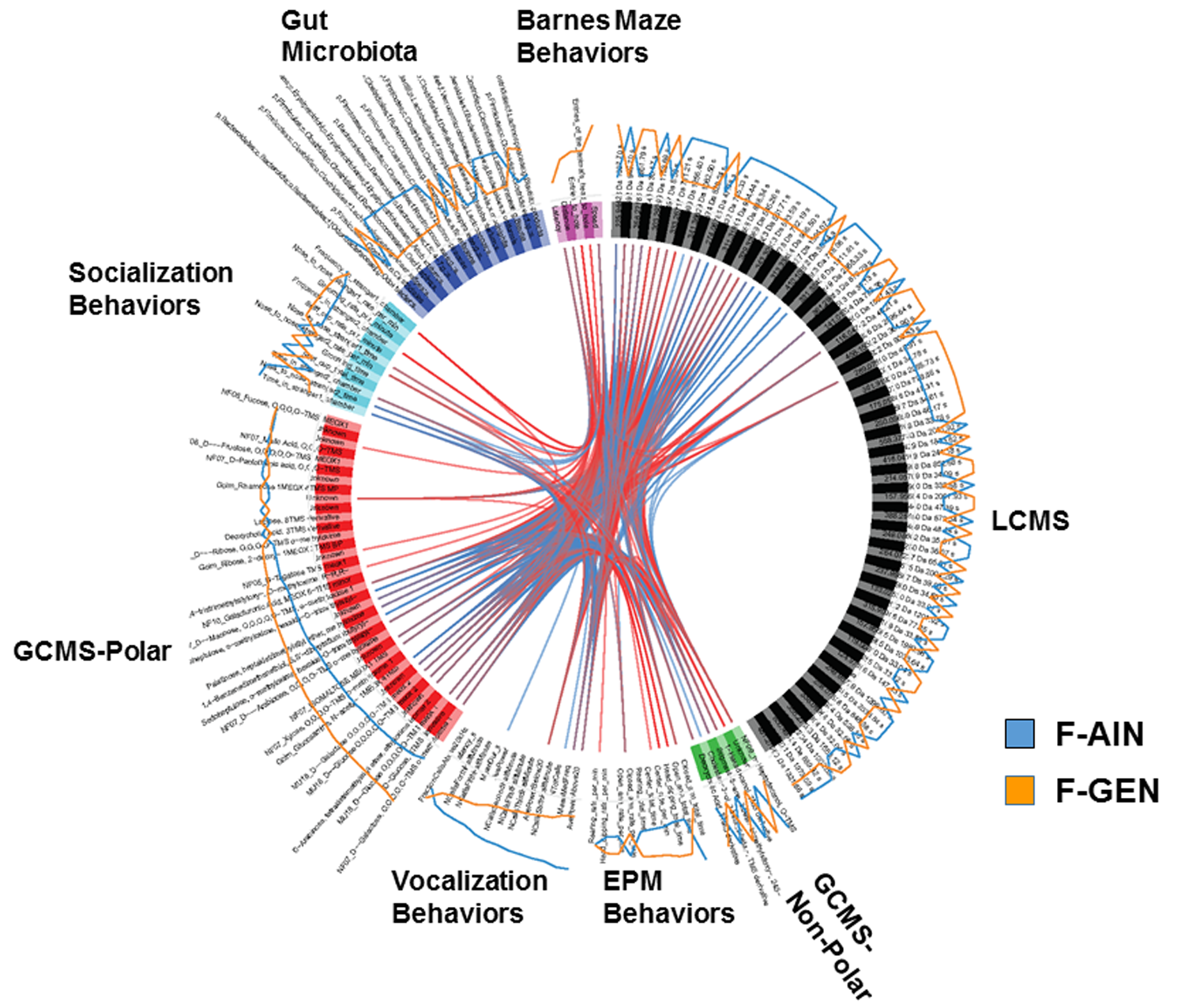


**Supplementary Figure 12.** Circos plot correlations between gut bacterial, fecal metabolome, and behavioral parameters in GEN females vs. AIN females. Red lines in the center indicate a positive correlation. In contrast, blue lines indicate a negative correlation. Results for AIN females are indicated with a blue line outside of the circle. Orange line indicates results for GEN females. The color of the line further from the circle indicates the treatment group where these results are greater.


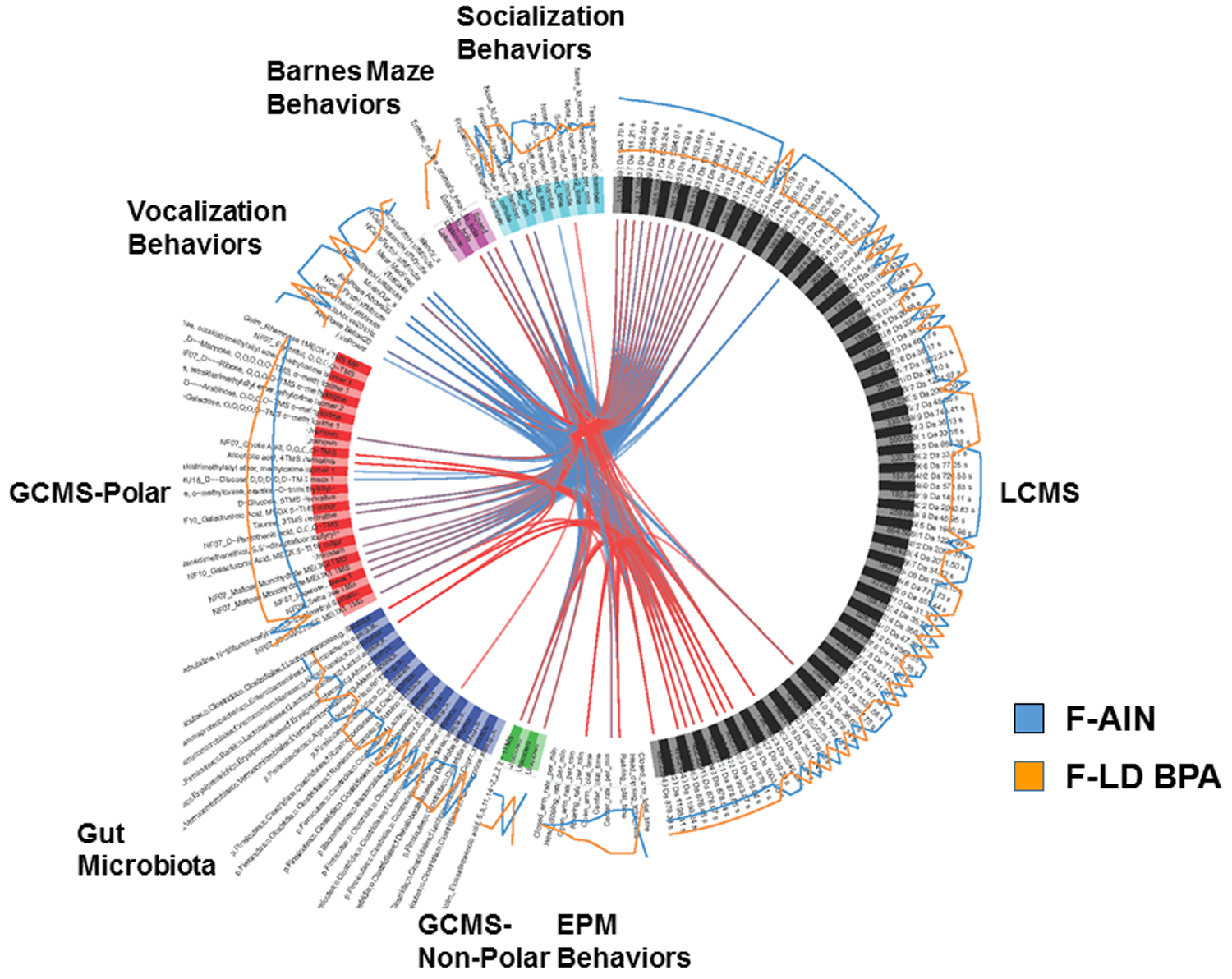


**Supplementary Figure 13.** Circos plot correlations between gut bacterial, fecal metabolome, and behavioral parameters in LD BPA females vs. AIN females. Red lines in the center indicate a positive correlation. In contrast, blue lines indicate a negative correlation. Results for AIN females are indicated with a blue line outside of the circle. Orange line indicates results for LD females. The color of the line further from the circle indicates the treatment group where these results are greater.

**
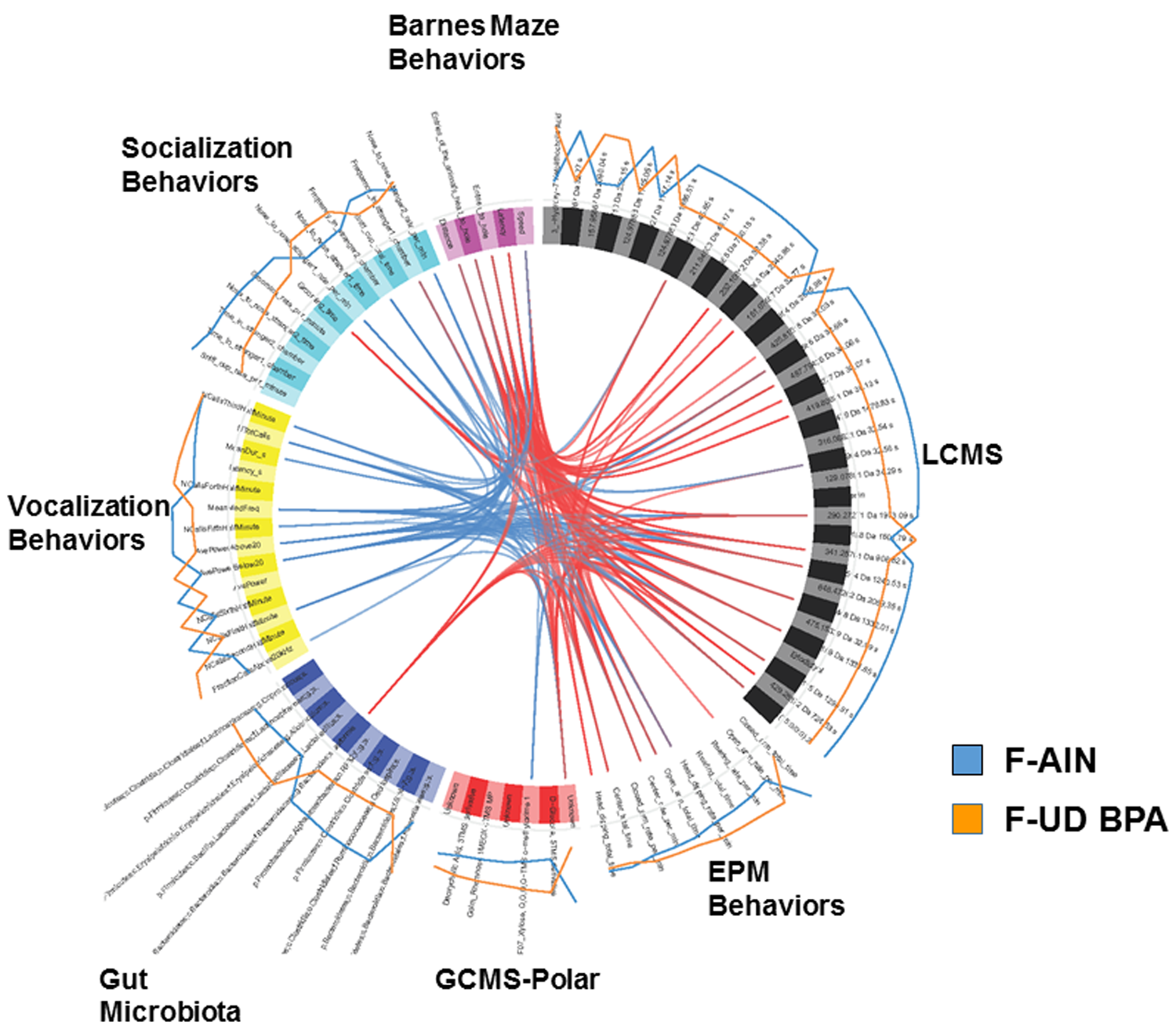
**

**Supplementary Figure 14.** Circos plot correlations between gut bacterial, fecal metabolome, and behavioral parameters in UD females vs. AIN females. Red lines in the center indicate a positive correlation. In contrast, blue lines indicate a negative correlation. Results for AIN females are indicated with a blue line outside of the circle. Orange line indicates results for UD BPA females. The color of the line further from the circle indicates the treatment group where these results are greater.


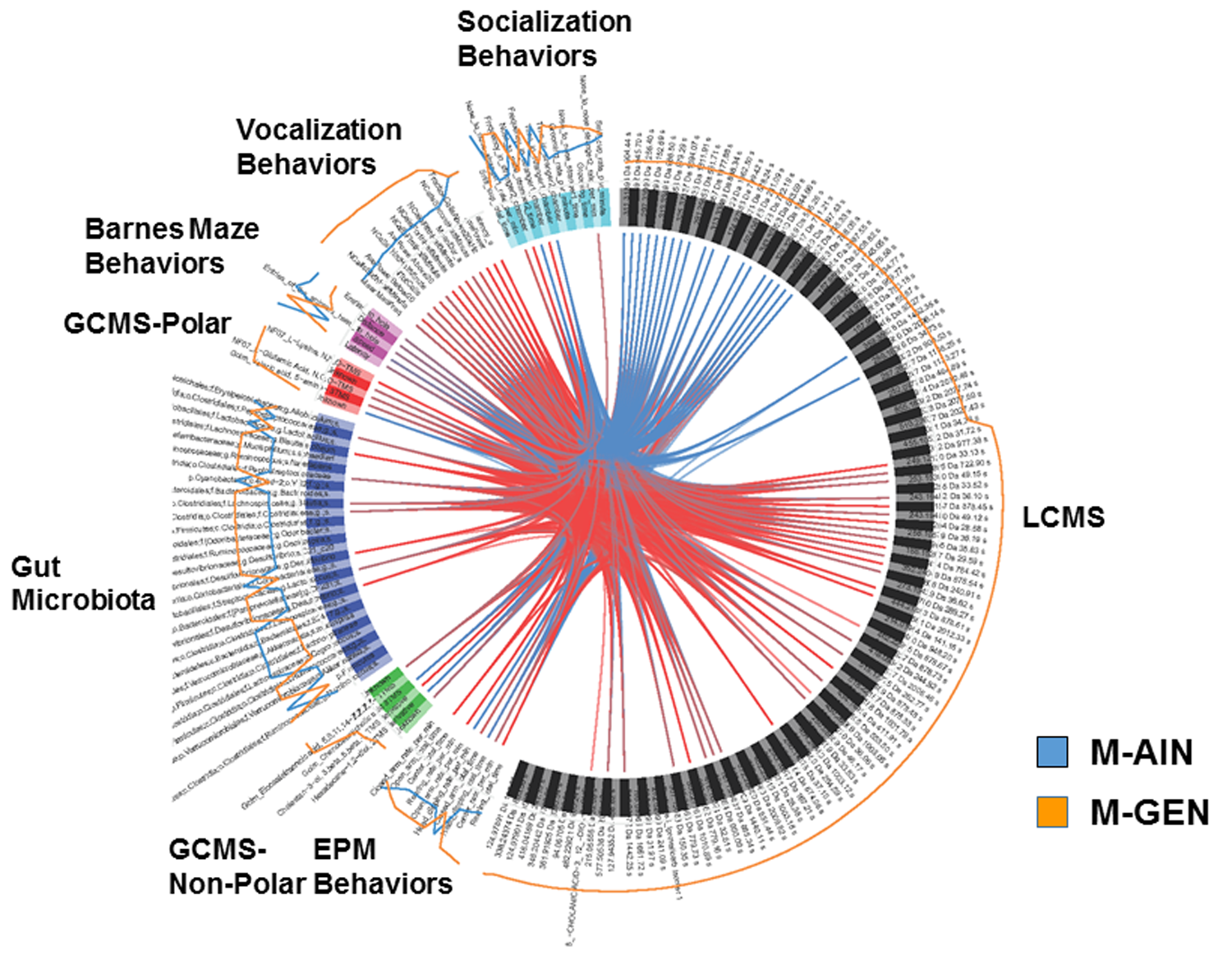


**Supplementary Figure 15.** Circos plot correlations between gut bacterial, fecal metabolome, and behavioral parameters in GEN males vs. AIN males. Red lines in the center indicate a positive correlation. In contrast, blue lines indicate a negative correlation. Results for AIN males are indicated with a blue line outside of the circle. Orange line indicates results for GEN males. The color of the line further from the circle indicates the treatment group where these results are greater.


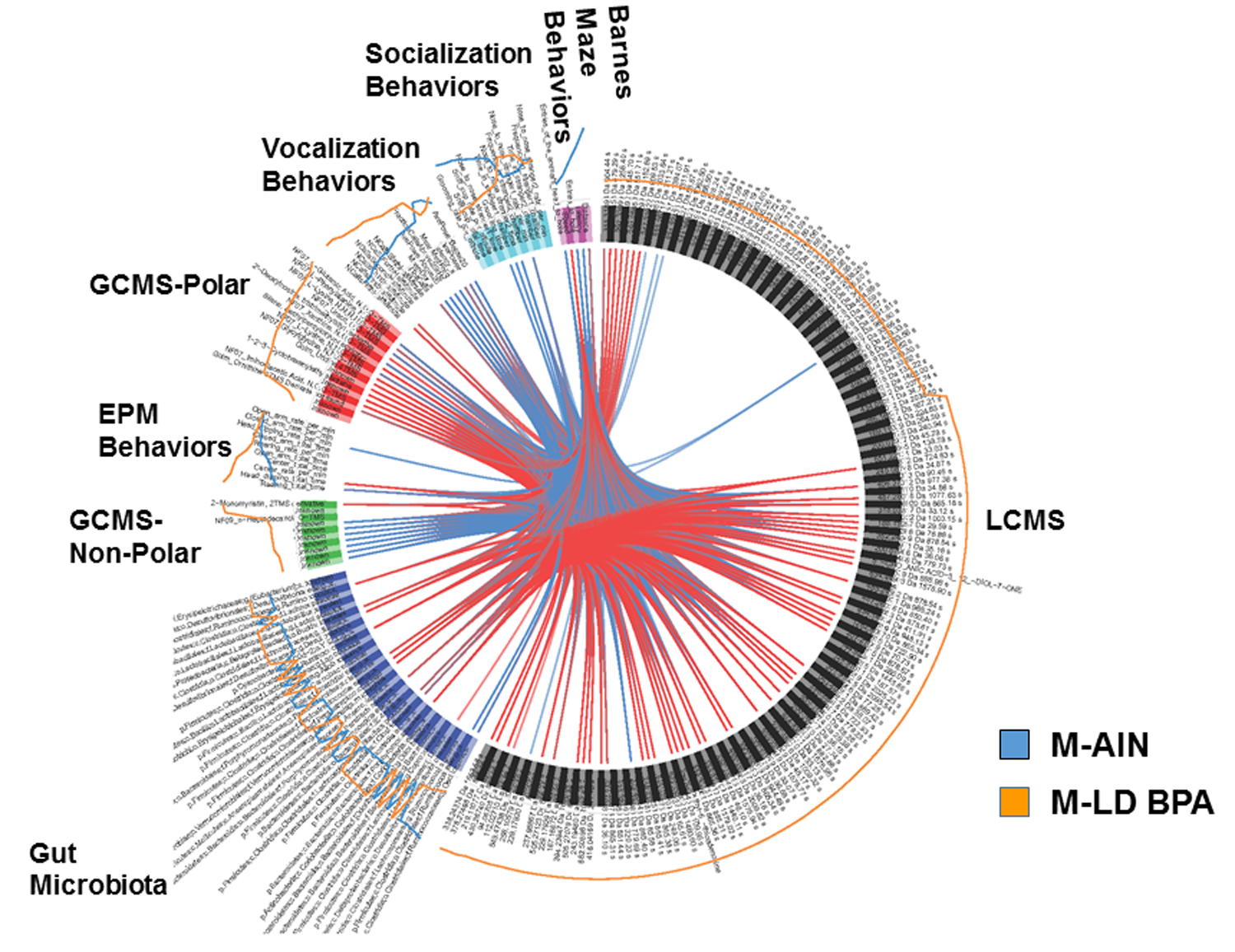


**Supplementary Figure 16.** Circos plot correlations between gut bacterial, fecal metabolome, and behavioral parameters in LD BPA males vs. AIN males. Red lines in the center indicate a positive correlation. In contrast, blue lines indicate a negative correlation. Results for AIN males are indicated with a blue line outside of the circle. Orange line indicates results for LD BPA males. The color of the line further from the circle indicates the treatment group where these results are greater.

**
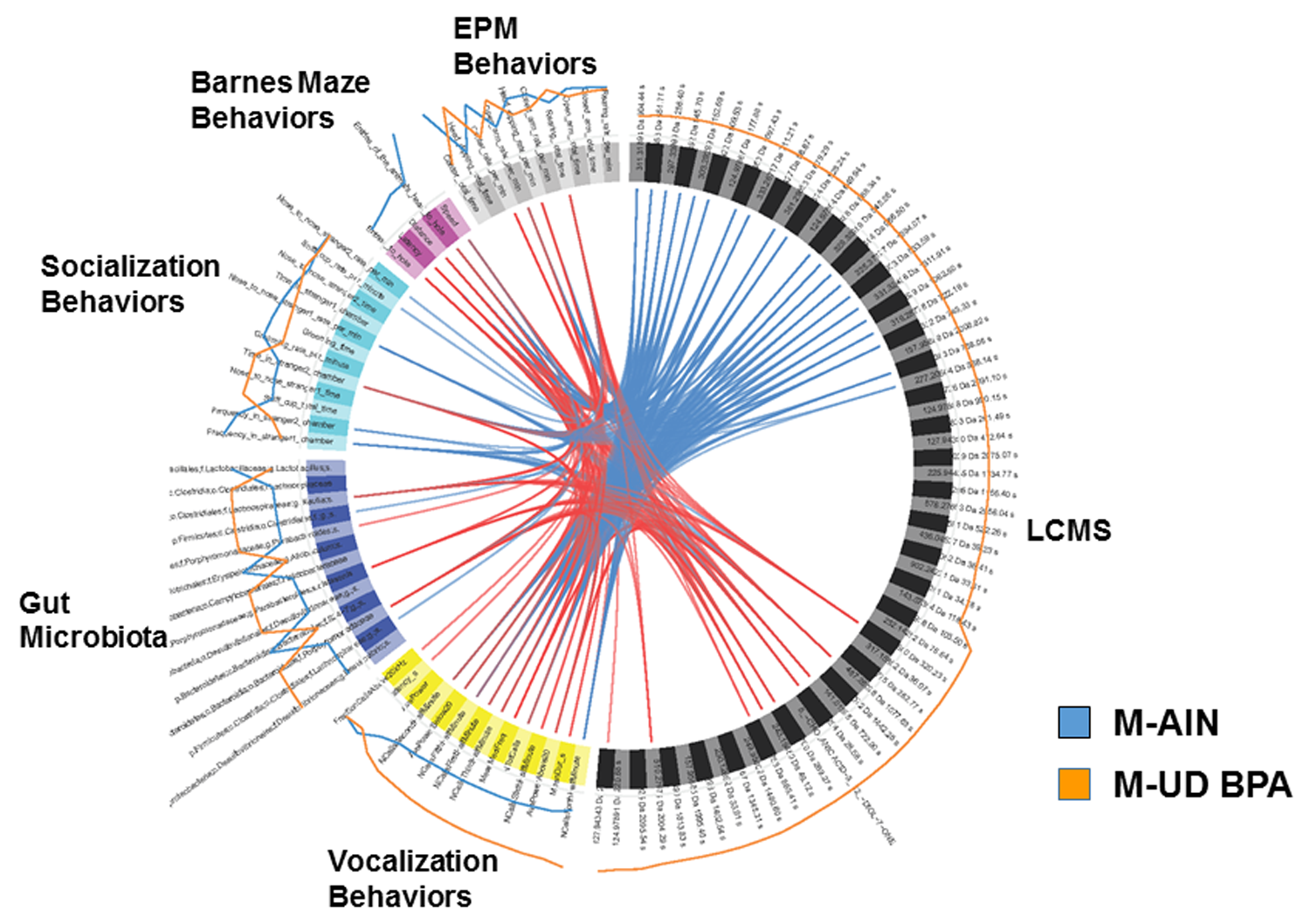
**

**Supplementary Figure 17.** Circos plot correlations between gut bacterial, fecal metabolome, and behavioral parameters in UD males vs. AIN males. Red lines in the center indicate a positive correlation. In contrast, blue lines indicate a negative correlation. Results for AIN males are indicated with a blue line outside of the circle. Orange line indicates results for UD BPA males. The color of the line further from the circle indicates the treatment group where these results are greater.

**Supplementary Tables**

**Supplementary Table 1.** Latency to enter the correct hole, average velocity, and duration of time spent in correct zone in the Barnes maze based on perinatal treatment and sex.

|  | **AIN** | | **GEN** | | **LD BPA** | | | **UD BPA** | | |
| --- | --- | --- | --- | --- | --- | --- | --- | --- | --- | --- |
| **Behavior** | **Female** | **Male** | **Female** | **Male** | | **Female** | **Male** | | **Female** | **Male** |
| **Duration of Time Spent in Correct Zone (seconds)** | 1.33 ± 0.35 | 1.48 ± 0.36 | 1.29 ± 0.63 | 0.48 ± 0.11 | | 2.12 ± 0.71 | 2.21 ± 1.16 | | 1.31 ± 0.28 | 2.81 ± 0.79 |
| **Latency to Enter Correct Hole (seconds)** | 173.8 ± 27.9 | 144.5 ± 26.2 | 135.9 ± 26.6 | 81.4 ± 11.2 | | 108.7 ± 20.4 | 140.5 ± 35.2 | | 180.5 ± 18.4 | 194.1 ± 28.4 |
| **Average Velocity (mm/seconds)** | 0.08 ± 0.01 | 0.11 ± 0.01 | 0.12 ± 0.01 | 0.14 ± 0.01 | | 0.12 ± 0.01 | 0.11 ± 0.02 | | 0.09 ± 0.01 | 0.10 ± 0.01 |

**Supplementary Table 2.** Duration of interactions with Stranger 2 mouse in Trial 3 of social testing and repetitive behaviors measured in the EPM based on perinatal treatment and sex

|  | **AIN** | | **GEN** | | **LD BPA** | | **UD BPA** | |
| --- | --- | --- | --- | --- | --- | --- | --- | --- |
| **Behavior** | **Female** | **Male** | **Female** | **Male** | **Female** | **Male** | **Female** | **Male** |
| **Trial 3 of Social Testing** | | | | | | | | |
| **Total Duration of Interaction with Stranger 1 (seconds)** | 119.3 ± 55.0 | 129.5 ± 36.7 | 167.3 ± 67.8 | 146.3 ± 39.3 | 73.6 ± 16.9 | 74.0 ± 14.1 | 91.4 ± 15.4 | 148.3 ± 64.7 |
| **Total Duration of Interaction with Stranger 2 (seconds)** | 174.8 ± 53.6 | 160.1 ± 40.9 | 77.5 ± 20.7 | 103.6 ± 21.4 | 145.7 ± 45.8 | 130.3 ± 40.3 | 127.7 ± 26.4 | 189.7 ± 53.7 |
| **Repetitive Behaviors Measured in EPM** | | | | | | | | |
| **Number of Times Rearing** | 77.0 ± 26.7 | 60.0 ± 15.4 | 127.6 ± 33.2 | 167.8 ± 28.2 | 164.3 ± 27.7 | 155.2 ± 26.4 | 113.1 ± 32.6 | 65.2 ± 19.4 |
| **Number of Times Engaging in Head Dipping Behavior** | 42.9 ± 11.3 | 39.8 ± 9.2 | 80.3 ± 18.2 | 92.0 ± 20.7 | 108.4 ± 25.9 | 89.1 ± 21.1 | 40.9 ± 11.8 | 56.0 ± 16.7 |

**Reference Cited in Supplementary Information**

1 Ma, J. *et al.* High-fat maternal diet during pregnancy persistently alters the offspring microbiome in a primate model. *Nature Commun.* **5**, 3889, doi:10.1038/ncomms4889 (2014).

2 Thorburn, A. N., McKenzie, C. I. & Shen, S. Evidence that asthma is a developmental origin disease influenced by maternal diet and bacterial metabolites. *Nature Commun.* **6**, 7320, doi:10.1038/ncomms8320 (2015).

3 Caporaso, J. G. *et al.* Global patterns of 16S rRNA diversity at a depth of millions of sequences per sample. *Proc. Natl. Acad. Sci. U. S. A.* **108 Suppl 1**, 4516-4522, doi:10.1073/pnas.1000080107 (2011).

4 Walters, W. A. *et al.* PrimerProspector: de novo design and taxonomic analysis of barcoded polymerase chain reaction primers. *Bioinformatics* **27**, 1159-1161, doi:10.1093/bioinformatics/btr087 (2011).

5 Javurek, A. B. *et al.* Effects of exposure to bisphenol A and ethinyl estradiol on the gut microbiota of parents and their offspring in a rodent model. *Gut Microbes* **7**, 471-485, doi:10.1080/19490976.2016.1234657 (2016).

6 Koestel, Z. L. *et al.* Bisphenol A (BPA) in the serum of pet dogs following short-term consumption of canned dog food and potential health consequences of exposure to BPA. *Sci. Total Environ.* **579**, 1804-1814, doi:10.1016/j.scitotenv.2016.11.162 (2017).

7 Magoc, T. & Salzberg, S. L. FLASH: fast length adjustment of short reads to improve genome assemblies. *Bioinformatics* **27**, 2957-2963, doi:10.1093/bioinformatics/btr507 (2011).

8 Edgar, R. C. Search and clustering orders of magnitude faster than BLAST. *Bioinformatics* **26**, 2460-2461, doi:10.1093/bioinformatics/btq461 (2010).

9 DeSantis, T. Z. *et al.* Greengenes, a chimera-checked 16S rRNA gene database and workbench compatible with ARB. *Appl. Environ. Microbiol.* **72**, 5069-5072, doi:10.1128/aem.03006-05 (2006).

10 Caporaso, J. G. *et al.* QIIME allows analysis of high-throughput community sequencing data. *Nature Methods* **7**, 335-336, doi:10.1038/nmeth.f.303 (2010).

11 Love, M. I., Huber, W. & Anders, S. Moderated estimation of fold change and dispersion for RNA-seq data with DESeq2. *Genome Biol.* **15**, 550, doi:10.1186/s13059-014-0550-8 (2014).

12 Chong, J. *et al.* MetaboAnalyst 4.0: towards more transparent and integrative metabolomics analysis. *Nucleic Acids Res.* **46**, W486-w494, doi:10.1093/nar/gky310 (2018).

13 Chong, J., Wishart, D. S. & Xia, J. Using MetaboAnalyst 4.0 for Comprehensive and Integrative Metabolomics Data Analysis. *Curr. Protocols Bioinformatics* **68**, e86, doi:10.1002/cpbi.86 (2019).

14 Marshall, B. L. *et al.* Early genistein exposure of California mice and gut microbiota-brain axis effects. *J. Endocrinol.*, doi:10.1530/joe-19-0214 (2019).

15 Tsugawa, H. *et al.* MS-DIAL: data-independent MS/MS deconvolution for comprehensive metabolome analysis. *Nature Methods* **12**, 523-526, doi:10.1038/nmeth.3393 (2015).

16 Rohart, F., Gautier, B., Singh, A. & Le Cao, K.-A. mixOmics: An R package for ‘omics feature selection and multiple data integration. *PLoS Comput. Biol.* **13**, e1005752 (2017).

17 González, I., Lê Cao, K.-A., Davis, M. J. & Déjean, S. Visualising associations between paired ‘omics’ data sets. *BioData Mining* **5**, 19 (2012).

18 McMurdie, P. J. & Holmes, S. phyloseq: an R package for reproducible interactive analysis and graphics of microbiome census data. *PLoS One* **8**, e61217, doi:10.1371/journal.pone.0061217 (2013).
